# The importance of understanding (pre)catalyst activation in versatile C–H bond functionalisations catalysed by [Mn_2_(CO)_10_]†

**DOI:** 10.1039/d4sc01215a

**Published:** 2024-05-03

**Authors:** Jonathan B. Eastwood, Thomas J. Burden, L. Anders Hammarback, Chris Horbaczewskyj, Theo F. N. Tanner, Ian P. Clark, Gregory Greetham, Michael Towrie, Ian J. S. Fairlamb, Jason M. Lynam

**Affiliations:** a Department of Chemistry, University of York York YO10 5DD UK ian.fairlamb@york.ac.uk jason.lynam@york.ac.uk; b Central Laser Facility, Research Complex at Harwell, STFC Rutherford Appleton Laboratory Harwell Campus Didcot Oxfordshire OX11 0QX UK

## Abstract

Mn-catalysed reactions offer great potential in synthetic organic and organometallic chemistry and the success of Mn carbonyl complexes as (pre)catalysts hinges on their stabilisation by strong field ligands enabling Mn(i)-based, redox neutral, catalytic cycles. The mechanistic processes underpinning the activation of the ubiquitous Mn(0) (pre)catalyst [Mn_2_(CO)_10_] in C–H bond functionalisation reactions is now reported for the first time. By combining time-resolved infra-red (TRIR) spectroscopy on a ps–ms timescale and *in operando* studies using *in situ* infra-red spectroscopy, insight into the microscopic bond activation processes which lead to the catalytic activity of [Mn_2_(CO)_10_] has been gained. Using an exemplar system, based on the annulation between an imine, 1, and Ph_2_C_2_, 2, TRIR spectroscopy enabled the key intermediate [Mn_2_(CO)_9_(1)], formed by CO loss from [Mn_2_(CO)_10_], to be identified. *In operando* studies demonstrate that [Mn_2_(CO)_9_(1)] is also formed from [Mn_2_(CO)_10_] under the catalytic conditions and is converted into a mononuclear manganacycle, [Mn(CO)_4_(C^N)] (C^N = cyclometallated imine), a second molecule of 1 acts as the oxidant which is, in turn, reduced to an amine. As [Mn(CO)_4_(C^N)] complexes are catalytically competent, a direct route from [Mn_2_(CO)_10_] into the Mn(i) catalytic reaction coordinate has been determined. Critically, the mechanistic differences between [Mn_2_(CO)_10_] and Mn(i) (pre)catalysts have been delineated, informing future catalyst screening studies.

## Abstract 2

Vibrational spectroscopy on time scales from picoseconds to hours unveils the activation pathways for the C–H bond functionalisation (pre)catalyst [Mn_2_(CO)_10_]. CO-loss, rather than Mn–Mn bond cleavage, give entry into a Mn(i)-based catalytic cycle.

## Graphical abstract

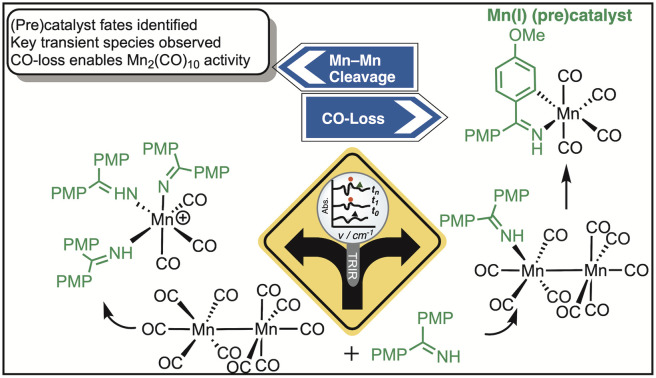

## Introduction

Transition metal catalysed C–H bond functionalisation reactions offer a low cost, chemo- and regio-selective method of achieving structural diversification.^1^ They enable the transformation of cheap hydrocarbon-based starting materials into valuable, versatile products while removing the need for reagent (pre)functionalisation, resulting in less waste with a concomitant higher atom efficiency than traditional cross-coupling reactions.^2^ There is a common consensus that a balance is required between the application of precious metal catalysts and earth abundant metal catalysts – the pros and cons for the use of either class are rather complicated, but important to understand.^3,4^ That said, it is incumbent on the chemistry community to remove the reliance on precious metals and enable wider applications of earth abundant metals.^5^ That will ultimately be achieved through broader applications of earth abundant metals (*i.e.* in industrial processes), a more holistic understanding of reaction sensitivities (*e.g.* O_2_, water, salts) and greater insight into the reaction mechanisms (*i.e.* (pre)catalyst activation, behaviour, and speciation events). Given the diverse reaction pathways available to 3d-metals,^6,7^ the mechanistic understanding of reactions catalysed by earth abundant metals lags behind that of precious metals, which can be complicated by fewer techniques being available for their study in standard laboratory settings. Using *in situ* spectroscopic techniques (*e.g.* IR, NMR, UV-vis) can be particularly effective for understanding the sequence of events involved in (pre)catalyst activation for precious metals and earth abundant metals, the subsequent catalyst and substrate turnover, and final deactivation pathway(s) for the active catalyst species.

We and others have been particularly inspired by the development and application of Mn-catalysed reactions, particularly those involving low oxidation state, stable d^6^/d^7^ Mn complexes, possessing strong field ligands such as CO.^8–10^ Stoichiometric C–H bond functionalisation reactions mediated by Mn-carbonyl compounds are well rooted in the history and development of the field of organometallic chemistry (going back to the 1960s). However, it was not until Kuninobu's breakthrough report of a Mn(i) carbonyl-catalysed insertion of aldehydes into C–H bonds in 2007,^11^ that a whole range of catalytic transformations have subsequently been reported following a similar mode of reactivity principles involving the Mn(i) centre. Primarily these have employed [MnBr(CO)_5_]^12^ or [Mn_2_(CO)_10_]^13^ as (pre)catalysts. The activity of [MnBr(CO)_3_(NCMe)_2_]^14^ and [Mn_2_Br_2_(CO)_8_]^15–18^ have been reported more recently.

**Fig. 1:**
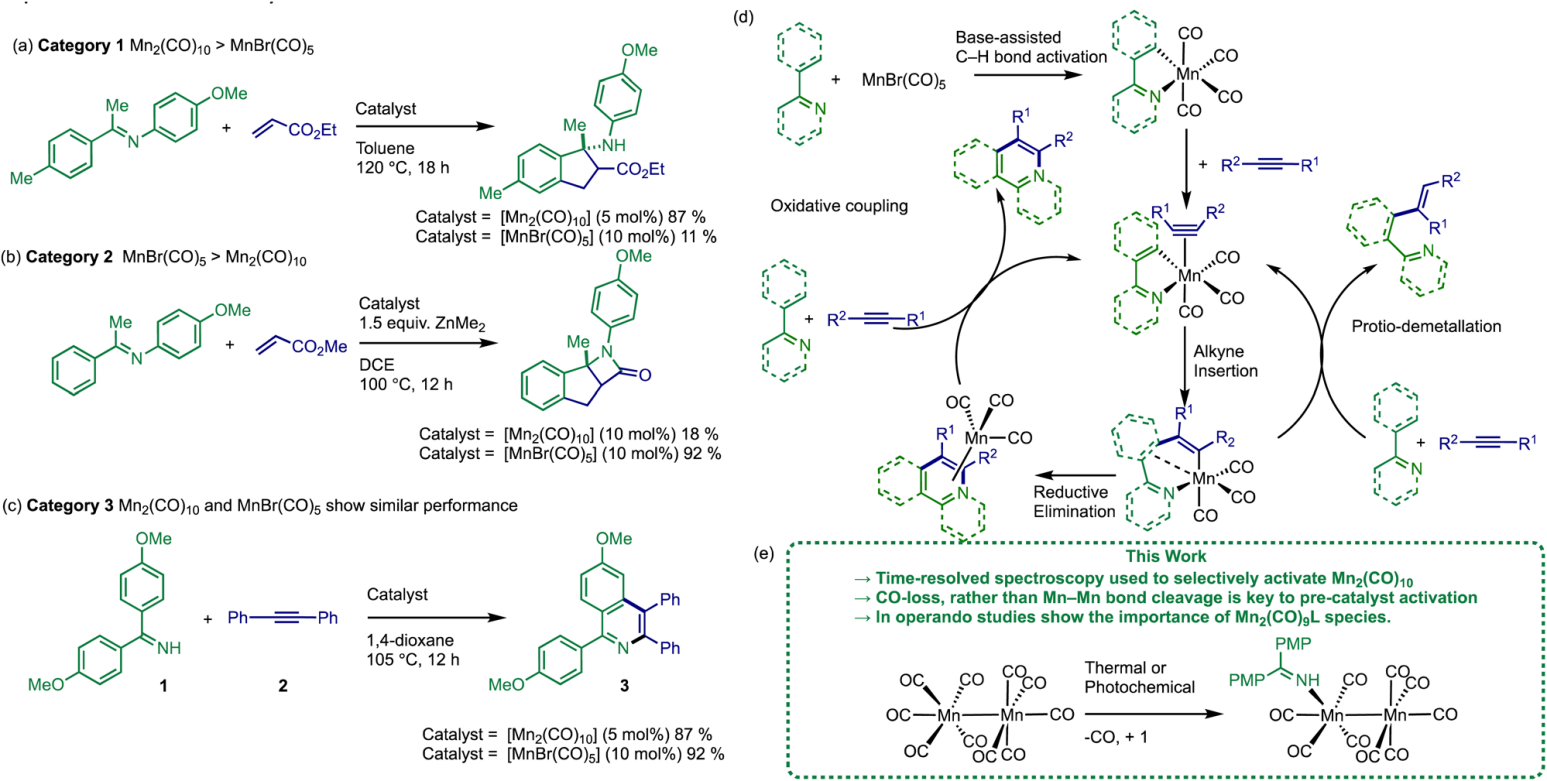
(a–c) Different classes of Mn-catalysed C–H bond functionalisation reactions. (d) Mn(i)-based mechanism for Mn-catalysed C–H bond functionalisation reactions. (e) Key finds from this work, PMP = (*p*-methoxyphenyl).

A survey of the literature reveals that the relative catalytic activity of [MnBr(CO)_5_] and [Mn_2_(CO)_10_] can be broken down into three different categories (Fig. 1). In Category 1 the use of [Mn_2_(CO)_10_] as (pre)catalyst results in significantly enhanced substrate conversion, when compared to [MnBr(CO)_5_] (see example Fig. 1a).^19–24^ The situation is reversed in Category 2 (Fig. 1b) where [MnBr(CO)_5_] exhibits a considerably higher yield when compared to [Mn_2_(CO)_10_].^12,25–29^ In Category 3, the performance of both (pre)catalysts is similar, Fig. 1c.^11,13,30–33^

The prevailing mechanistic hypothesis for the (pre)catalysts, is that the catalytic cycle proceeds through a series of Mn(i) species. The reactions are proposed to be isohypsic (redox neutral) with a core d^6^ low-spin *fac*-Mn(CO)_3_ structural unit enabling the key steps of C–H bond activation, migratory insertion with a seven-membered manganacycle acting as an anvil point on the reaction coordinate (Fig. 1d).^34–39^

The (pre)catalyst activation of [MnBr(CO)_5_] in the alkenylation of 2-phenylpyridine (as a model reaction) was examined. Two routes of (pre)catalyst activation were discovered involving either direct activation of the C–H bond on 2-phenylpyridine or, if this is slow, then a terminal alkyne could be preferentially manganated to give an Mn(i)-alkynyl complex, in a reaction reminiscent of the Sonogashira cross-coupling involving Cu(i)-alkynyl species.^40^ These data support a global mechanistic hypothesis based on a common Mn(i) cycle.

To date the pathways leading to the activation of [Mn_2_(CO)_10_] in C–H bond functionalisation reactions has not been delineated. For [Mn_2_(CO)_10_] to enter the common reaction pathway exhibited by Mn(i) (pre)catalysts, oxidation from Mn(0) to Mn(i) is formally required, a critical point which has been ignored by the field thus far. Although the possibility of a different mechanistic pathway for the [Mn_2_(CO)_10_]-catalysed reactions cannot be excluded. Establishing the nature of the (pre)catalyst activation for [Mn_2_(CO)_10_] is vital to explain the differences in reactivity between the commonly employed Mn-carbonyl (pre)catalysts.

To gain mechanistic insight into the activation of [Mn_2_(CO)_10_], the previously reported dehydrogenative [4 + 2] annulation reaction of imine, 1, and alkyne, 2, to give isoquinoline 3 was selected as an exemplar system (Fig. 1c).^13^ This reaction belongs to Category 3, exhibiting similar results when mediated by either [MnBr(CO)_5_] or [Mn_2_(CO)_10_] (92% and 87% respectively), when performed at identical [Mn] loadings.

IR spectroscopy was selected as the primary method of monitoring chemical change at the metal centre, as the changes to the intensity and band positions of the vibrational modes of the coordinated carbonyl ligands provides information about changes to the geometry and electronic environment of the manganese complex. A two-pronged approach was used to evaluate (pre)catalyst activation and *in operando* activity. In the first instance, time-resolved spectroscopy (picosecond–millisecond timescale) was used to explore the two divergent reaction pathways available for [Mn_2_(CO)_10_], these studies were complemented by *in operando* (second-hour timescale) reaction monitoring of [Mn_2_(CO)_10_] and a range of other potential catalyst systems.

## Results and discussion

As a (pre)catalyst, [Mn_2_(CO)_10_] only has two primary activation pathways: either Mn–Mn bond cleavage to give two [Mn(CO)_5_] radicals,^41^ or CO-dissociation to give [Mn_2_(CO)_9_].^42^ The [Mn(CO)_5_] radical may recombine to reform [Mn_2_(CO)_10_],^43,44^ but in the presence of suitable donor ligands, L, it may be oxidised to form [Mn(CO)_5_L]^+^ or [Mn(CO)_3_L_3_]^+^.^45–47^ Coordinatively unsaturated [Mn_2_(CO)_9_] may also bind a donor ligand to form [Mn_2_(CO)_9_L].^48,49^ The photochemistry of [Mn_2_(CO)_10_] offers an almost unique opportunity to selectively access these different reaction pathways, as irradiation at 400 nm results predominantly in the formation of [Mn(CO)_5_]. By contrast, excitation at higher energy results in CO-loss and formation of [Mn_2_(CO)_9_].^42^ Therefore, selective excitation at either wavelength will enable the only two possible intermediates, in either photochemical or thermal reactions, to be directly accessed. A strategy was envisaged in which performing these experiments in the presence of the substrate for the catalytic reaction will then enable the fate of the different Mn reaction intermediates to be determined.

Complementary experiments were then designed in which the catalytic reaction would be monitored *in operando* using IR spectroscopy. This would then enable a link between the time-resolved spectroscopic measurements and the nature of the species formed under working conditions to be made. It is a combination of these approaches that provides insight into the processes underpinning [Mn_2_(CO)_10_]-catalysed reactions.

### Time-resolved spectroscopy

**Fig. 2:**
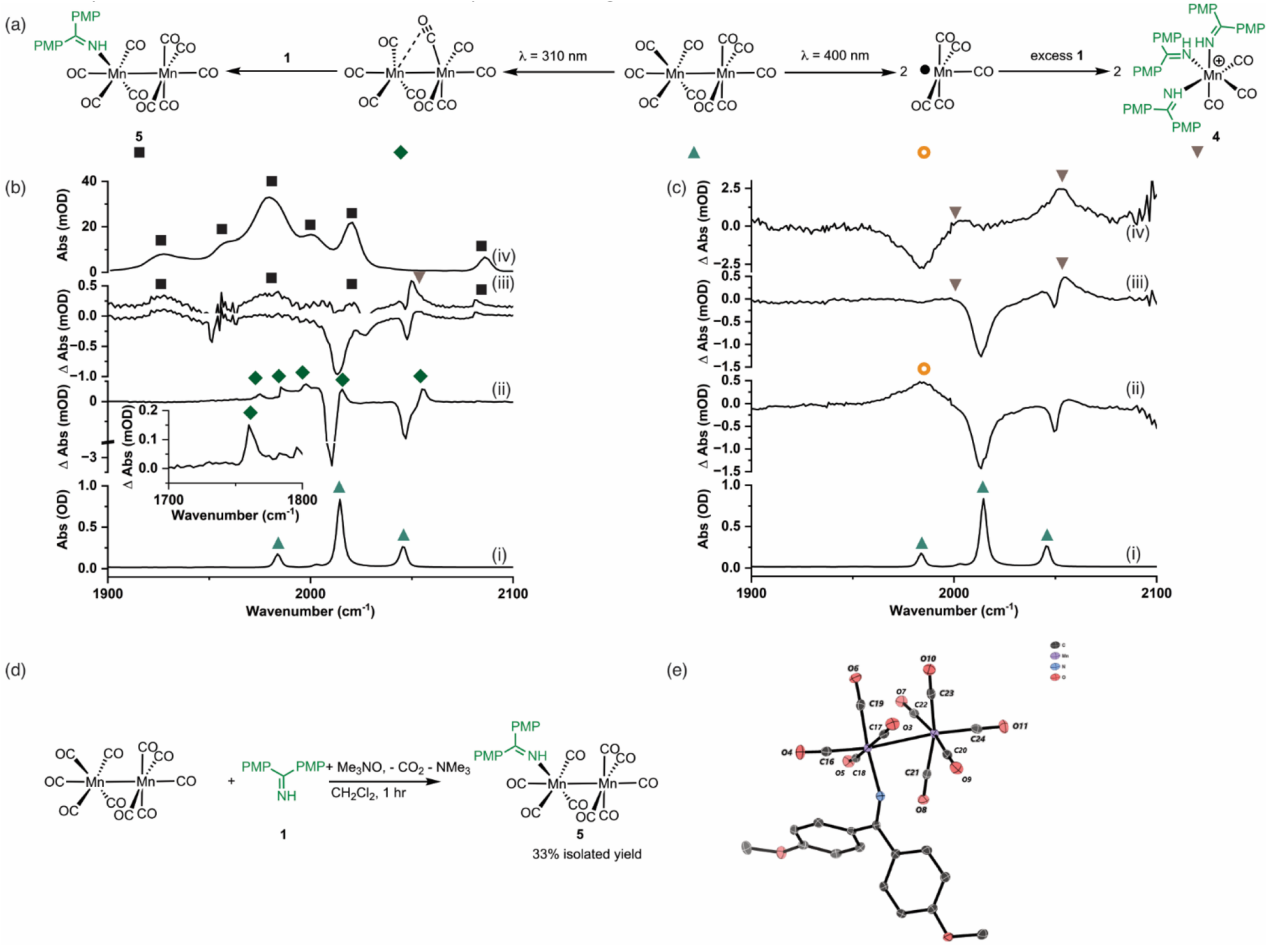
(a) Reaction scheme showing the fate of [Mn_2_(CO)_10_] following photolysis at *λ* = 310 nm (left) and *λ* = 400 nm (right). (b) (i) FTIR spectrum of [Mn_2_(CO)_10_]. (ii) TRIR difference spectrum (*λ* = 310 nm) of a sample of [Mn_2_(CO)_10_] and 1 recorded with a pump–probe delay of 1 ns showing the formation of [Mn_2_(CO)_9_]. (iii) difference spectrum of a sample of [Mn_2_(CO)_10_] and 1 recorded with a pump–probe delay of 20 μs showing the formation of 5. The spectrum above has had the bleach bands for [Mn_2_(CO)_10_] subtracted. (iv) FTIR spectrum of 5. (c) (i) FTIR spectrum of [Mn_2_(CO)_10_]. (ii) TRIR difference spectrum (*λ* = 400 nm) of a sample of [Mn_2_(CO)_10_] and 1 recorded with a pump–probe delay of 1 μs showing the formation of [Mn(CO)_5_]. (iii) difference spectrum of a sample of [Mn_2_(CO)_10_] and 1 recorded with a pump–probe delay of 20 μs showing the formation of 4. (iv) Difference spectrum of a sample of [Mn_2_(CO)_10_] and 1 recorded with a pump–probe delay of 20 μs showing the formation of 4 with the bleach bands for [Mn_2_(CO)_10_] subtracted. (d) Synthetic route to compound 5. (e) Molecular structure of 5 from single crystal X-ray diffraction analysis with hydrogen atoms removed for clarity. Thermal ellipsoids are shown at the 50% probability level.

Time-resolved multiple probe spectroscopy (TR^M^PS) was used to study the interaction of these photoproducts towards imine 1 over a temporal range of one picosecond to several 100 microseconds. As seen in previous studies,^41^ excitation of [Mn_2_(CO)_10_] at 400 nm in toluene solution resulted in predominantly Mn–Mn bond cleavage (72%) (based on the intensity of the bleach band recovery) and the characteristic band for [Mn(CO)_5_] was observed at 1984 cm^−1^. The remaining 28% of the photoproduct was [Mn_2_(CO)_9_] q.v. (Fig. 2c(ii)). In the absence of the imine 1, radical–radical recombination was observed over the course of *ca.* 200 μs; [Mn_2_(CO)_10_] reformed with a second order rate constant of (1.04 ± 0.03) × 10^9^ mol^−1^ dm^3^ s^−1^. A related experiment in the presence of imine 1 resulted in faster loss (*ca.* 30 μs) of [Mn(CO)_5_] with the concomitant formation of a new band at 2055 cm^−1^ with a further peak superimposed on the bleach band for [Mn_2_(CO)_10_] at 1984 cm^−1^ (Fig. 2c(iii)). These features remained for the duration of the experiment. The appearance of these two bands is consistent with the formation of a new complex with *C*_3v_ symmetry. The number and position of the bands is consistent with the formation of a complex *fac*-[Mn(CO)_3_(L)_3_]^+^, where L is a nitrogen donor ligand. For example *fac*-[Mn(CO)_3_(NCMe)_3_]^+^ has bands at 2063.0 and 1973.7 cm^−1^.^50^ Therefore this species was assigned to *fac*-[Mn(CO)_3_(1)_3_]^+^, 4,^50,51^ and its formation mirrors the reported reactivity of [Mn(CO)_5_] towards N-donor-containing ligands.^46,52^

Excitation of [Mn_2_(CO)_10_] at 310 nm in toluene solution resulted in photochemically induced CO loss, forming the known complex [Mn_2_(CO)_9_] with a distinctive semi-bridging CO ligand possessing a stretching mode at 1764 cm^−1^ (Fig. 2b(ii)).^49^ In the absence of imine 1, the photoproduct remained for the duration of the experiment whereas in the presence of imine 1 the bands for [Mn_2_(CO)_9_] (including the peak at 1764 cm^−1^) disappeared over the course of *ca.* 50 μs and peaks for a new species assigned as [Mn_2_(CO)_9_(1)], 5 (Fig. 2b(iii)), was observed over the grew in intensity with an observed rate constant of (2.1 ± 0.7) × 10^5^ s^−1^.

The identification of 5 was secured by the preparation of an authentic sample formed by reaction of [Mn_2_(CO)_10_] with 1 in the presence of Me_3_NO (Fig. 2d)^53^ – the latter activates CO loss through formation of CO_2_ and NMe_3_. The resulting complex was isolated and characterised by NMR and IR spectroscopy as well as single-crystal X-ray diffraction (Fig. 2e). Notably the IR spectrum of the authentic sample (Fig. 2b(iv)) was in excellent agreement with the assigned bands in the TRIR spectrum.

The results from the TR^M^PS experiments therefore demonstrated that in the presence of 1, Mn–Mn bond cleavage of [Mn_2_(CO)_10_] leads to *fac*-[Mn(CO)_3_(1)_3_]^+^, 4, *via* [Mn(CO)_5_], whereas CO-loss from [Mn_2_(CO)_10_] leads to [Mn_2_(CO)_9_(1)], 5, through [Mn_2_(CO)_9_]. Importantly, light-generated [Mn_2_(CO)_9_] did not exhibit any interactions with PhC_2_Ph, excluding the role of the alkyne in (pre)catalyst activation.

### *In operando* studies

**Fig. 3:**
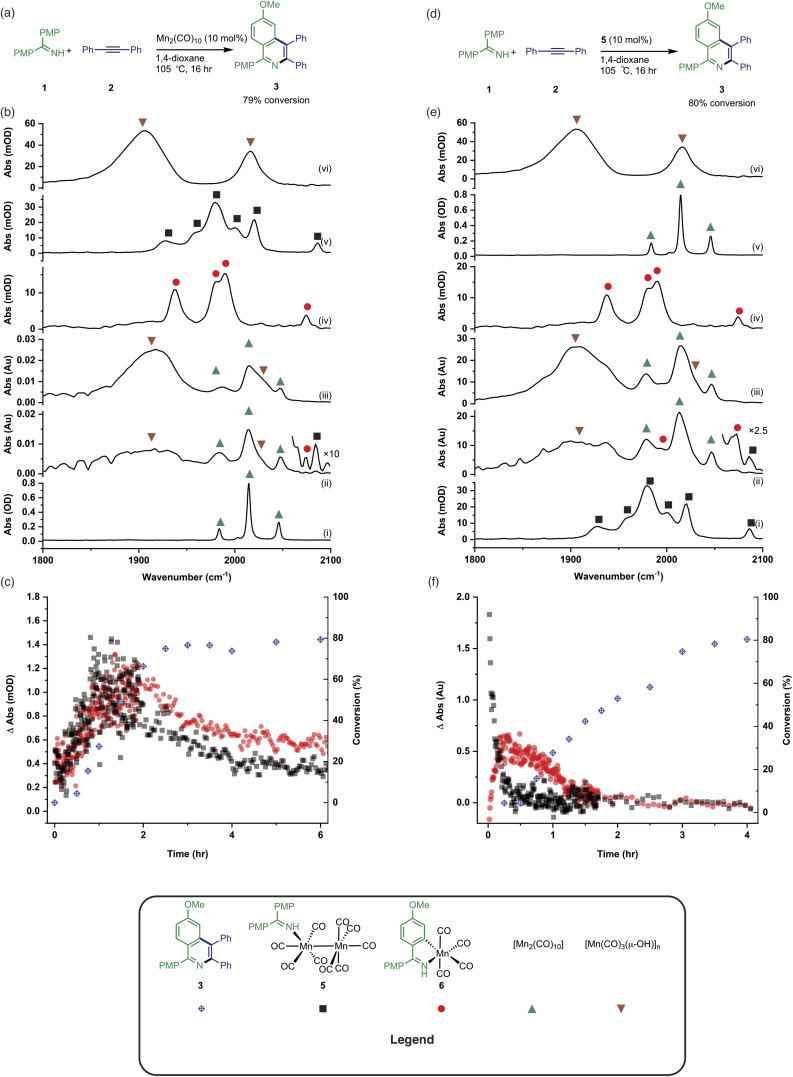
(a) Reaction scheme showing the formation of 3 catalysed by [Mn_2_(CO)_10_]. (b) (i) FTIR spectrum of [Mn_2_(CO)_10_]. (ii) *In situ* IR spectrum recorded using the conditions in (a) after 2 hours. (iii) *In situ* IR spectrum recorded using the conditions in (a) after 6 hours. (iv) FTIR spectrum of 6. (v) FTIR spectrum of 5. (vi) FTIR spectrum of [Mn_7_(μ_3_-OH)_8_(CO)_18_]. (c) Kinetic profiles showing the formation and loss of 5 and 6 when compared to conversion to 3 using the conditions in (a). (d) Reaction scheme showing the formation of 3 catalysed by 5. (e) (i) FTIR spectrum of 5. (ii) *In situ* IR spectrum recorded using the conditions in (d) after 20 min. (iii) *In situ* IR spectrum recorded using the conditions in (d) after 1 hour. (iv) FTIR spectrum of 6. (v) FTIR spectrum of [Mn_2_(CO)_10_]. (vi) FTIR spectrum of [Mn_7_(μ_3_-OH)_8_(CO)_18_]. (f) Kinetic profiles showing the formation and loss of 5 and 6 when compared to conversion to 3 using the conditions in (d).

Having established the two divergent pathways in which [Mn_2_(CO)_10_] interacts with 1, the reaction between 1 and 2 to form 3 (catalysed by [Mn_2_(CO)_10_]) was monitored through *in situ* IR experiments using a Mettler-Toledo ReactIR silicon-tipped ATR-IR probe (Fig. 3a). Firstly, two minor bands grew in at 2074 and 2085 cm^−1^ (Fig. 3b), which occurred prior to the formation of 3. Given this temporal profile, it is likely these complexes are involved in the (pre)catalyst activation pathway. The bands at 2074 and 2085 cm^−1^ showed a good match to authentic samples of [Mn_2_(CO)_9_(1)] 5 (Fig. 3b(iv)) and the known, catalytically competent,^13^ manganacycle 6 (Fig. 3b(v)) respectively.

The formation of 3 exhibited a sigmoidal kinetic profile (open diamonds, Fig. 3c) further supported the hypothesis that a complex route of (pre)catalyst activation was occurring. Secondly, the bands belonging to [Mn_2_(CO)_10_] persisted for the duration of the experiment, indicating that either [Mn_2_(CO)_10_] is regenerated, or a small proportion of the (pre)catalyst ‘leaches’ into an active form. Finally, beyond *ca.* 3 hours the spectra were dominated by a broad feature centred at 1917 cm^−1^ (Fig. 3b(iii)) which corresponds to known unreactive manganese(i) hydroxy-bridged clusters (Fig. 3b(vi)) which have been detected in catalytic reaction mixtures when reagents have been consumed and side reactions with residual water outcompete catalytic turnover.

When 5 was used as a (pre)catalyst under the standard reaction conditions, compound 3 was obtained in 88% yield, however, *fac*-[Mn(CO)_3_(NCMe)_3_]^+^ (which was used as a model for 4) exhibited no activity. Taken together with the *in situ* IR data, this indicates that the initial activation proceeds through CO-loss from [Mn_2_(CO)_10_] to give [Mn_2_(CO)_9_(1)], 5, which then subsequently forms 6.

An experiment was then carried out using 5 as the (pre)catalyst and monitored *via in situ* IR spectroscopy. The bands belonging to 5 depleted over the course of *ca.* 30 minutes with an observed rate constant of (2.7 ± 0.6) × 10^−3^ s^−1^. In their place, bands at 1979, 1993, 2012, 2017, 2046, and 2072 cm^−1^ grew in intensity, along with further bands positioned at lower wavenumber (1850–1950 cm^−1^). The bands at 1979, 1993, 2072 cm^−1^ and an unresolved feature at *ca.* 1940 cm^−1^ grew in with an observed rate constant of (2.7 ± 0.6) × 10^−3^ s^−1^. By comparison with an authentic sample, these peaks were assigned to manganacycle 6, in which the imine 2 had undergo a C–H bond activation. The bands at 1979, 2012, and 2046 cm^−1^ closely align with those for [Mn_2_(CO)_10_] in 1,4-dioxane solution, and grew in with an observed rate constant of (1.9 ± 0.1) × 10^−3^ s^−1^. This indicated that on heating 5 undergoes Mn–Mn bond cleavage to form both 6 and [Mn(CO)_5_], the latter then undergoes a dimerisation to give the observed [Mn_2_(CO)_10_]. The formation of 6 indicates that one Mn in 5 must be oxidized to Mn(i) and rapidly undergo C–H bond activation.

**Fig. 4:**
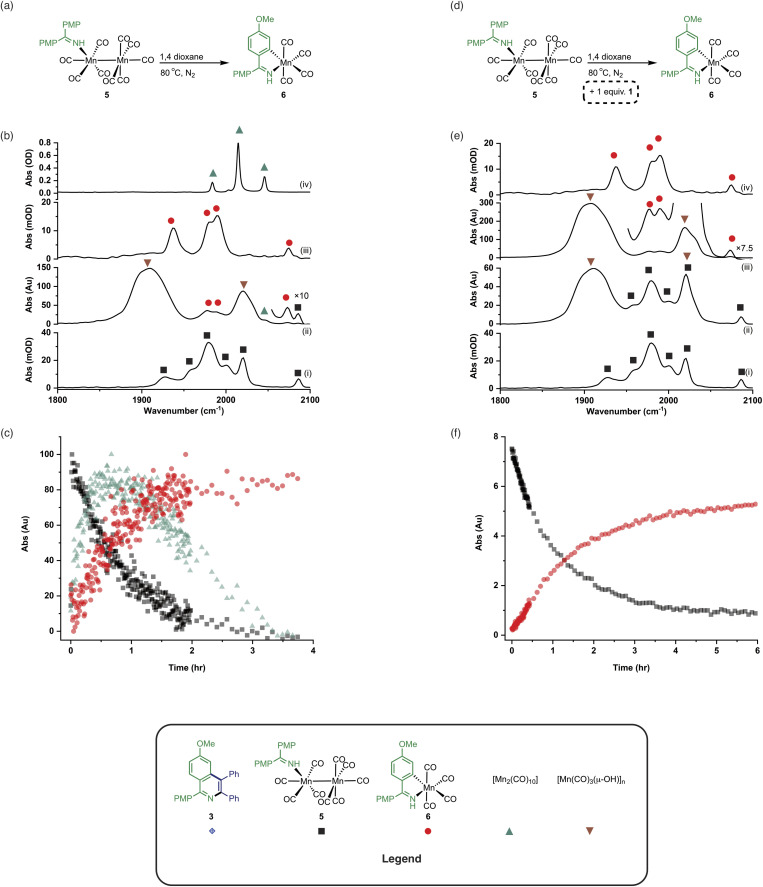
(a) Reaction showing the formation of 6 from 5. (b) (i) FTIR spectrum of 5. (ii) *In situ* IR spectrum recorded using the conditions in (a) after 3 hours. (iii) FTIR spectrum of 6. (iv) FTIR spectrum of [Mn_2_(CO)_10_]. (c) Kinetic profiles showing the formation and loss of [Mn_2_(CO)_10_], 5 and 6 using the conditions in (a). (d) Reaction showing the formation of 6 from 5 in the presence of added 1. (e) (i) FTIR spectrum of 5. (ii) *In situ* IR spectrum recorded using the conditions in (d) after 1.5 hours. (iii) *In situ* IR spectrum recorded using the conditions in (d) after 4.5 hours. (iv) FTIR spectrum of 6. (f) Kinetic profiles showing the loss of 5 and formation of 6 using the conditions in (d).

The observation of the formation of 6 is important as complexes of this type are well-established intermediates in Mn-catalysed reactions and 6 may act as a (pre)catalyst in its own right, coupling 1 and 2 to give 3 with 95% yield.^13^ Therefore, the route of C–H bond activation which presumably leads from 5 to 6 was investigated. Involvement of internal alkyne 2 in this process could be excluded as no pathway to form an alkynyl complex is viable. A sample of 5 was heated at 80 °C and monitored *via in situ* IR spectroscopy. The bands due to 5 were observed to deplete over *ca.* 4 hours with the initial formation of [Mn_2_(CO)_10_] and inactive hydroxy-bridged Mn clusters.^40,54^ After a slight lag-time from the detection of [Mn_2_(CO)_10_], bands due to the formation of 6 were observed (Fig. 4b(ii)).

These data indicated that uncoordinated 1 may be playing a role in the C–H bond activation process as it was postulated that heating 5 resulted in small amounts of decomposition to give [Mn_2_(CO)_10_] (as observed) and 1. The reaction was repeated in the presence of one equivalent of 1. The vibrational modes associated with 5 depleted (Fig. 4c(ii)) with an observed rate constant of (2.0 ± 0.1) × 10^−4^ s^−1^ while those for 6 grew in with a statistically identical rate constant (2.2 ± 0.2) × 10^−4^ s^−1^ (Fig. 4e). In this instance, formation of [Mn_2_(CO)_10_] prior to formation of 6 did not occur, supporting the hypothesis that uncoordinated 1 is required to enable C–H bond activation.

**Fig. 5:**
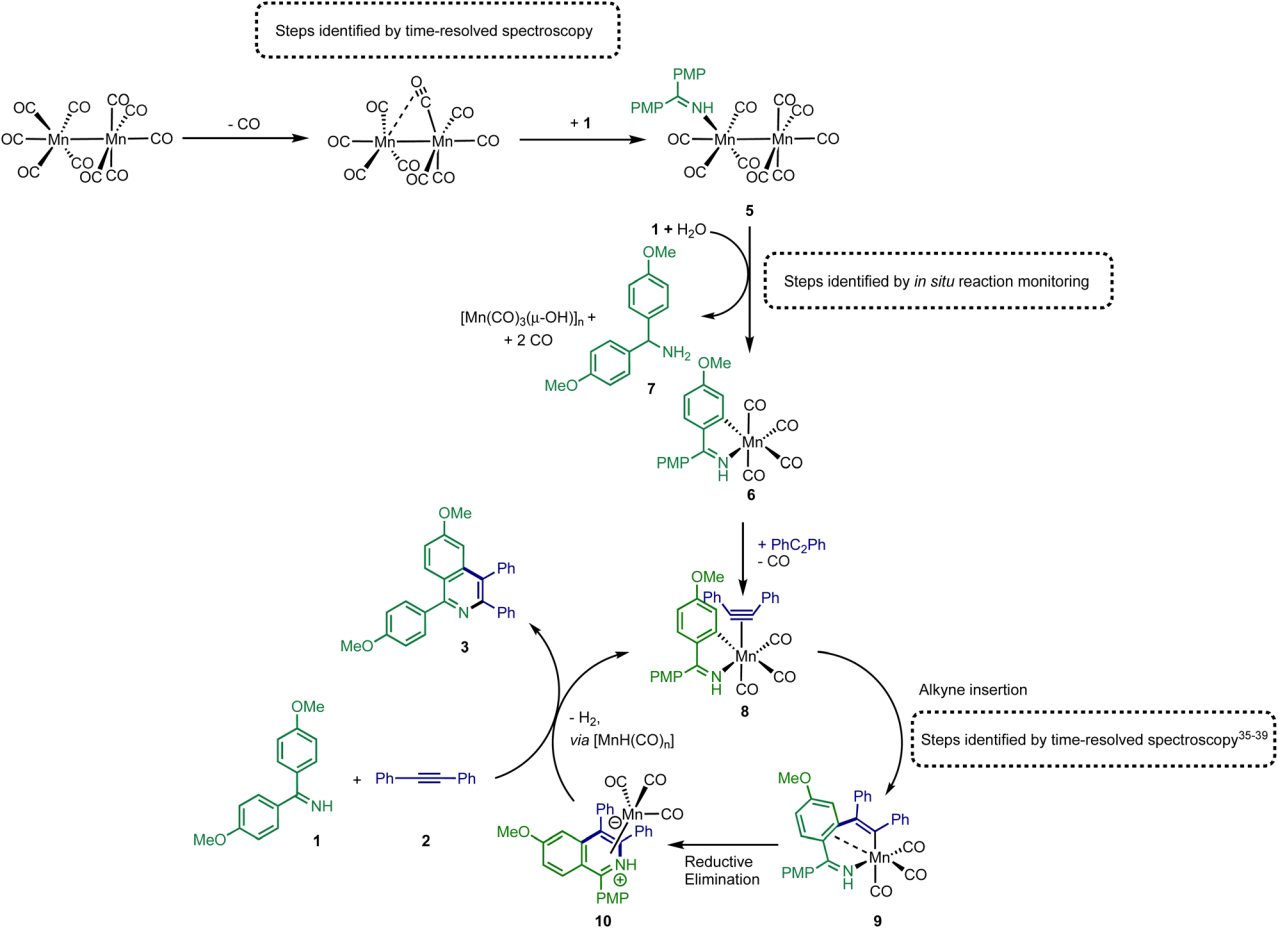
Proposed route for (pre)catalyst activation of [Mn_2_(CO)_10_] and subsequent catalytic cycle.

Insight into the role played by imine 1 in this process was obtained from analysis of ^1^H NMR spectra recorded on reaction aliquots for the conversion of 1 and 2 to 3 catalysed by 5. The spectra revealed the presence of amine 7 which is derived from imine 1 (Fig. 5). It is therefore proposed that free 1 can play two roles in (pre)catalyst activation. Firstly, 1 is a sacrificial oxidant, being reduced to amine 7, balancing the overall redox state as the manganese is oxidized to Mn(i). Secondly amine 7 will act as a stronger base than free imine aiding with the C–H bond activation required to form 6 and potentially mimicking the role played by NCy_2_H in other Mn-catalysed reactions.

## Conclusions

The combination of the data obtained from time-resolved spectroscopy and *in situ* studies on catalytic reactions provides detailed insight into pathways leading to activation of [Mn_2_(CO)_10_] under catalytic conditions. As shown in Fig. 5, the first step in the catalyst activation pathways is loss of CO from [Mn_2_(CO)_10_] and coordination of 1 to give complex 5. Subsequent reaction of 5 with 1 results in the formation of amine 7 and manganacycle 6. The spectroscopic data provides evidence for the concomitant formation of manganese-cluster compounds, [Mn(CO)_3_(μ-OH)]_*n*_ which, it is proposed, is the fate of the “Mn(CO)_5_” fragment of the dimer 5 during conversion to 6. This is consistent with the net two-electron oxidation of 5 where water acts as the hydrogen source to reduce 1 to 7. Based on the proposed mechanism, water is only required for catalyst initiation, not turn-over. The water content of the solvent used in the reactions was 20 ppm (Karl Fischer analysis) which corresponds to 20 mol% with respect to manganese, which may explain why not all the [Mn_2_(CO)_10_] present is consumed in the reaction (Fig. 3b(iii)). A large excess of water, however, completely inhibited catalysis with only the formation of the hydroxide-bridged clusters being observed.

These transformations provide a route to 6, which has already been shown to be catalytic competent,^13^ and a direct route into a Mn(i)-mediated catalytic cycle has been uncovered (Fig. 5). In this catalytic cycle, CO loss from 6 occurs enabling the formation of alkyne complex 8. The alkyne then undergoes migratory insertion into the Mn–C bond to give 9, a step which we have previously quantified by time-resolved spectroscopy.^10,35,37–39^ A formal C–N reductive elimination would then give 10 and in model studies we have identified that such species can be formed from the reaction of metallacycles with alkynes.^34,38,40^ Finally, the product, 3 can be eliminated with the formal elimination of H_2_. The original paper reporting the formation of 3 from 1 and 2 suggested that manganese hydride complexes are generated in this step^13^ which can then promote the C–H bond activation in 1 to regenerate a metalated intermediate *en route* to 8. Although no evidence for the formation of [MnH(CO)_5_], for example, was obtained from our *in operando* mechanistic studies, we cannot exclude its formation and putative hydride intermediates provide a viable mechanistic pathway.

The data reported in the manuscript provide a readily explanation for the performance in Category 3 reactions (Fig. 1) in which [Mn_2_(CO)_10_] and [MnBr(CO)_5_] show essentially identical behaviour as a direct pathway to 6 from [Mn_2_(CO)_10_] has been established and 6 (and related manganacycles) have been directly accessed from [MnBr(CO)_5_] in the presence of base.^12,13^ Therefore a common intermediate is formed from both (pre)catalysts and similar catalyst behaviour would be expected.

The requirement for the sacrificial oxidant provides an explanation for the observed difference in reactivity between [MnBr(CO)_5_] and [Mn_2_(CO)_10_] in Category 1 reactions. In reactions where a reagent can act as a sacrificial oxidant, [Mn_2_(CO)_10_] is able to readily form the catalytically competent 5-membered metallacycle, 6. However, if the reagents are unable to promote an initial cyclomanganation reaction with [MnBr(CO)_5_] then no activity is observed. This provides an explanation for the behaviour in Category 1 reactions (Fig. 1a) in which the performance of [Mn_2_(CO)_10_] is superior to [MnBr(CO)_5_].

In Category 2 (Fig. 1a) reactions, activation of [MnBr(CO)_5_] proceeds by initial metalation by ZnMe_2_, to give [MnMe(CO)_5_] which is then able to directly cyclomanganate the imine, or donor atom-containing, substrate proving a route into the catalytic cycle.^26^ Presumably under these conditions, it is not possible to oxidize the manganese centre in [Mn_2_(CO)_10_] to manganese(i). Therefore the 5-membered metallacycle is not accessible and reactivity is inhibited.

In summary, these results demonstrate a pathway for [Mn_2_(CO)_10_] to undergo activation *via* CO-loss, substrate coordination and subsequent oxidation. They also reinforce the key role played by 5-membered manganacycles, such as 6 in promoting Mn-catalysed C–H bond functionalisation reactions.

## Data availability

The data assoicated with this study are available at https://doi.org/10.15124/3a9da5b7-5b57-451a-80fc-09deee5473f9.

## Author contributions

JBE prepared compounds 1 and 5, performed the *in operando* spectroscopic measurements, acquired and processed the TRIR spectra. TJB and LAH assisted with the acquisition of the TRIR spectra and contributed to the scientific discussion on [Mn_2_(CO)_10_] chemistry. CH assisted with the acquisition of the TRIR spectra. TFNT collected and refined the X-ray data for compound 5. ICP performed the instrumental set-up and assisted with the data collection of the TRIR experiments on the LIFEtime instrument built and maintained by GG and MT. IJSF and JML conceived the project, envisaged the nature of the experiment design and data interpretation. IJSF and JML secured the funding for the programme of research underpinning all the results presented in the paper. JML prepared the manuscript with input from all co-authors.

## Conflicts of interest

There are no conflicts to declare.

## Supplementary Material

SC-015-D4SC01215A-s001

SC-015-D4SC01215A-s002
