# Supplementary material for: The importance of understanding (pre)catalyst activation in versatile C–H bond functionalisations catalysed by [Mn2(CO)10]
Source: Chem Sci. 2024 May 3;15(24):9183–91. doi: 10.1039/d4sc01215a (PMC11186345; doi:10.1039/d4sc01215a)
Supplement: SC-015-D4SC01215A-s001 [file SC-015-D4SC01215A-s001.pdf]

Electronic Supplementary Material (ESI) for Chemical Science.
This journal is © The Royal Society of Chemistry 2024

Supporting Information

The importance of understanding (pre)catalyst activation in versatile C–H bond functionalisations catalysed by [$Mn_2(CO)_{10}$]

Jonathan B. Eastwood,[a] Thomas J. Burden,[a] L. Anders Hammarback,[a] Chris Horbaczewskyj,[a] Theo F. N. Tanner,[a] Ian P. Clark,[b] Gregory Greetham,[b] Michael Towrie,[b] Ian J. S. Fairlamb,[a]* and Jason M. Lynam[a]*

[a] Department of Chemistry, University of York, Heslington, York, UK.

[b] Central Laser Facility, STFC Rutherford Appleton Laboratory, Harwell Campus, Didcot, Oxfordshire, UK

Contents

1. General information and procedures S2

2. Characterisation data S6

3. NMR Spectra S12

4. IR Spectra S21

5. Uv-Vis Spectra of $Mn_2(CO)_{10}$ used in Pump-Probe TRIR experiments S23

6. Additional control TRIR experiments S24

7. X-ray data S27

8. References S29

1. General information and procedures

Solvents and Reagents

Commercial chemicals were purchased from Acros Organics, Alpha Aesar, Apollo Scientific, Fisher Scientific, Fluorochem, Insight Biotechnology, Merck Life Science, Sigma-Aldrich, Strem Chemicals UK, or Tokyo Chemical Industry UK and were used without further purification unless otherwise stated. Dry MeOH, heptane and 1,4-dioxane were purchased from Acros Organics, stored over 4 Å molecular sieves and under an atmosphere of N_2. Dry Et_2O, hexane, MeCN, methylene chloride, THF and toluene were collected from a Pure Solv MD-7 solvent system and stored in oven dried ampoules under an atmosphere of N_2.

Petroleum ether (pet ether) in following experimental procedures refers to the distillate with a boiling point of 40−60 °C.

Room-temperature (RT) typically refers to 21 °C, with an upper and lower limit of 16−23 °C recorded.

Chromatography

Thin-layer chromatography (TLC) was conducted using Merck aluminium-backed 5554 silica plates. Visualisation of spots was achieved *via* irradiation (254 nm), or sequential staining with potassium permanganate followed by heating. Flash column chromatography was carried out following the procedure reported by Still *et al.*,[1] using Fluorochem silica gel 60 (particle size 40−63 µm), with the solvent system stated in the specific procedure.

Melting Points

Melting points were recorded on a Stuart digital SMP3 machine, with a temperature ramp of 2 °C min^{-1} used.

Infrared Spectroscopy

Infrared spectra for characterisation were obtained using a Unicam Research Series FTIR (Solution IR) or a Bruker APLHA-Platinum FTIR Spectrometer with a platinum−diamond ATR sampling module.

In situ IR spectroscopic measurements were made on a Mettler Toledo ReactIR ic10 with a K6 conduit SiComp (silicon) probe and MCT detector. An oven dried 3-necked round-bottom flask

equipped with a stirrer bar was attached to the ReactIR ic10 probe. One neck was sealed with a septum, and the other connected to a Schlenk line. The system was evacuated and subsequently backfilled with N_2 five times. Following this a background spectrum was recorded under an N_2 atmosphere. An internal thermocouple and dry deoxygenated solvent were introduced *via* the septum. The system was heated using a stirrer hotplate until thermocouple was giving steady readings at the desired temperature.

Sample measurements were then started at 30 second intervals, and reagents added in a sequential order. First any imine reagents were added, followed by unsaturated reagents, additives and finally any manganese containing compounds. Liquids were injected through the septum, whereas solids required rapid removal of the septum under a strong positive flow of N_2, addition, and replacement of the septum. After each addition, a comment was added to the experimental run, and at least 2 minutes were given for the IR signal/ reaction temperature to stabilise. A sampling interval of 30 seconds was maintained for the initial 2 hours of an experiment. This was extended to 5-minute intervals beyond the first 2 hours.

Peaks of interest between ~1800–2200 cm^{-1} with a resolution of ± 4 cm^{-1} were individually monitored on an experiment-by-experiment basis. A solvent subtraction, one- or two-point baseline and baseline offset were applied to all peaks. Where multiple peaks were convoluted, second derivative function was finally applied. Following this, peak data were exported into a Microsoft Excel document, and subsequent analysis conducted in OriginPro 2019b (64-bit) 9.6.5.169 (Academic) software.

UV–Visible spectroscopy

UV–Visible spectra were recorded with a Jasco V–560 spectrometer using Quartz cuvettes.

Nuclear Magnetic Resonance Spectroscopy

Solution phase ^{1}H and ^{13}C NMR analysis were carried out on a Brucker AV500 spectrometer (500 and 125 MHz for ^{1}H and ^{13}C respectively) at 298 K (295 K for methylene chloride). ^{13}C NMR spectra were recorded with ^{1}H decoupling. Spectra were processed in MestReNova software version 14.0.0-23239. In ^{1}H spectra, coupling constants were quoted with ± 0.5 Hz. Chemical shifts are reported in ppm and referenced to the residual non-deuterated solvent.

Residual $CDHCl_2$ in methylene chloride-d_2: ^{1}H: 5.32 ppm, ^{13}C: 53.49 ppm

Residual $CHCl_3$l in chloroform-d: ^{1}H: $CHCl_3$ 7.26 ppm, ^{13}C: $CHCl_3$ 77.36 ppm

^{1}H NMR peaks are reported to two decimal places, whereas ^{13}C are reported to one decimal place.

Mass Spectrometry

ESI MS spectra were measured using a Bruker Daltronics micrOTOF MS, Agilent series 1200LC with electrospray ionization. Liquid Injection Field Desorbtion Ionisation (LIFDI) mass spectrometry was carried out using a Waters GCT Premier MS Agilent 7890A GC. Data were quoted as a mass to charge ratio (m/z) in Daltons and relative intensity in parenthesis. High resolution mass spectra (HRMS) are reported within 5 ppm error of the theoretical value unless stated otherwise.

Single Crystal X-Ray Diffraction

Single crystals were crystalised from a suitable solvent system noted in the relevant synthetic procedure. An appropriate crystal was selected and [oil on 200 micrometre micromount] on a SuperNova, Dual, Cu at home/near, Eos diffractometer. The crystal was kept at 110.00(10) K during data collection. Using Olex2[2] , the structure was solved with the SHELXT[3] structure solution program using Intrinsic Phasing and refined with the SHELXL[4] refinement package using Least Squares minimisation.

Time-Resolved Multiple Probe Spectroscopy (TR^{M}PS)

TRIR measurements were carried out at the LIFEtime facility using TR^{M}PS technique at the Central Laser Facility (Science and Technology Facility Council Rutherford Appleton Laboratories).[5,6] The experiments were driven by a 100 kHz repetition rate Yb:KGW amplifier (Pharos) as a pump source, producing 15 W, 260 fs pulses at 1030 nm. The laser output was used to drive a BBO-based 515 nm pumped optical parametric amplifier (OPA). The pump beam was collimated, travelled along an programable optical delay line (0-16 ns 1200 mm long double pass), then focused onto the sample. The probe beam sources from a 100 kHz repetition rate YB:KGW amplifier(Pharos) producing 6W, 180 fs pulses at 1030 nm, driving two 3 W BBO/KTA based OPAs. The two Pharos sources shared a 80 MHz oscillator, allowing pump-probe delay steps of 12.5 ns. The probe beam was split to provide probe and reference pulses. The probe beams were collimated, synchronised by a fixed optical delay, and focused by a gold parabolic mirror onto the sample. The three beams were overlapped on the sample

using a 50 µm pinhole. The probe beams were measured by two separate 128-element detectors. To go beyond 12.5 ns, subsequent seed pulses can be selected from the 80 MHz oscillator. Data were collected using pump-probe delays ranging from 1 ps to 988.5 µs.

Samples were prepared as follows. To an oven dried amberised Duran bottle approx. 15 mg of manganese complex were added and dissolved in 20 mL of anhydrous solvent from a newly opened sure-seal bottle. The system was then sparged (N_2, Ar, or air) for 10 minutes with solution pumping around the system. For the duration of the experiment, the Duran flask was sealed while under a positive pressure of sparge gas. The Duran bottle was connected *via* PTFE tubing to a Harrick cell with a spacer (100 µm unless stated otherwise), with solvent being pumped round the system using a peristaltic pump. During experiments, the Harrick cell was attached to rastering, to prevent excitation of photoproducts. Following an experiment all solution was pumped from the system, 3×10 mL of new solvent pumped around to clean the kit, and dried using a positive pressure of N_2 for 10 minutes.

Initially spectra were processed in ULTRA_VIEW_v2 where negative times were subtracted, and a polynomial second order baseline correction was applied, and data exported as a csv file. The resulting data were then analysed in OriginPro 2019b (64-bit) 9.6.5.169 (Academic) software. Where data sets were particularly noisy, early time points after the first trump were deleted and up to a 20-point average of data points applied. Kinetic fits were performed with appropriate ExpGro, ExpDec and ExpGroDec functions and values were quoted with in the format of XX ± XX indicating the 95% confidence limits of values obtained from exponential fits. The suitability of kinetic fits were then assessed using the built in residual plots produced by OriginPro software.

2. Characterisation data

1,1-*bis*-(4-methoxyphenyl)methanimine (1)

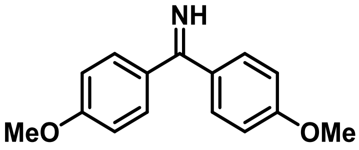

To an oven dried Schlenk equipped with a magnetic stirrer, under an N_2 atmosphere, was added 0.5 M 4-methoxyphenylmagnesium bromide (1 eq., 16.6 mL, 8.3 mmol). In a separate oven dried Schlenk, 4-methoxybenzonitrile (1 equ., 1.1 g, 8.3 mmol) was added and dissolved in anhydrous deoxygenated THF (6 mL). The 4-methoxybenzonitrile solution was added dropwise to the Grignard solution and heated to reflux overnight. The reaction mixture was cooled in an ice bath, quenched with anhydrous methanol (3 mL) and stirred for 30 minutes. The solution was diluted with 20 mL of hexane and filtered through a Celite® plug. The Celite® plug was washed with toluene (3×10 mL) and the filtrate reduced in *vacuo*. The pale yellow solid was recrystalised from methylene chloride/pentane to give a white crystalline solid being 1,1-*bis*-(4-methoxyphenyl)methanimine (1.75 g, 88 %).

m.p. = 132.8-133.2 ^{o}C. ^{1}H NMR ($CDCl_3$, 500 MHz)δ 7.56 (4H, d, *J* = 8.6 Hz), 6.93 (4H, d, *J* = 8.6 Hz), 3.85 (6H, s). ^{13}C NMR ($CDCl_3$, 125 MHz) δ 177.3, 161.7, 131.4, 130.7, 113.6, 55.5. ESI-MS m/z (ion, %): 242.1176 ($[M+H]^+$, 100). IR (hexane, cm^{-1}): 2969 (w), 2963 (w), 2995 (w), 2940 (w), 2926 (w), 2901 (w), 2888 (w), 2878 (w), 2845 (w), 2360 (w), 2341 (w), 1607 (m), 1514 (m), 1476 (w), 1390 (w), 1360 (m), 1295 (w), 1250 (s), 1172 (m) and 1038 (m).

6-Methoxy-1-(4-methoxyphenyl)-3,4-diphenylisoquinoline (3)

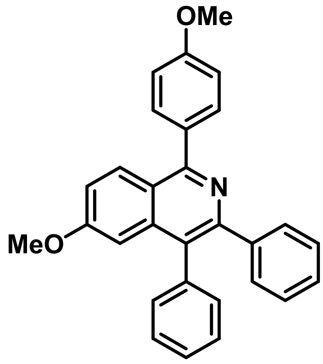

To an oven dried Schlenk that was evacuated, backfilled with N_2 five times and equipped with a magnetic stirrer, 1,1-*bis*-(4-methoxyphenyl)methanimine (1 eq., 1 mmol, 241 mg), diphenyl acetylene (1.5 eq., 1.5 mmol, 267 mg) and $[Mn_2(CO)_{10}]$ (0.1 eq., 0.1 mmol, 27.5 mg) were added. Anhydrous deoxygenated toluene (10 mL) was added and the reaction stirred at 105 ^oC for 6hr. Once complete, solvent was removed in *vacuo* and the product purified by flash column chromatography (94:6 *v/v* pet ether: diethyl ether). to give a white powder (345 mg, 83 %).

^1H NMR (500 MHz, $CDCl_3$) δ 8.13 (1H, d, J = 8.6 Hz), 7.77 (2H, m), 7.44-7.34 (5H, m), 7.32-7.28 (2H, m), 7.22-7.14 (4H, m), 7.10-7.06 (2H, m), 6.97, (1H, d, *J* = 8.6 Hz), 3.91, (3H, s), 3.74 (3H, s). ^{13}C NMR ($CDCl_3$, 125 MHz) δ 160.62,160.10, 158.95, 150.34, 141.25, 139.23, 138.04, 132.60, 131.64, 131.38, 129.65, 128.84, 128.52, 127.60, 127.34, 127.03, 121.31, 118.89, 113.88, 104.31, 55.55, 55.35. R_f 0.25 (94:6 *v/v* pet ether: diethyl ether). ESI- MS m/z (ion, %) 418 ($[M+H]^+$, 100 %). IR (Hexane, cm^{-1}): 3097 (w), 3077 (w), 3041 (m), 3029 (w), 2933 (w), 2921 (w), 2886 (w), 2835 (w), 1615 (m), 1578 (w), 1567 (w), 1542 (w), 1513 (w), 1497 (w), 1487 (w), 1454 (w), 1441 (w), 1410 (w), 1381 (w), 1339 (w), 1291 (w), 1263 (w), 1247 (m), 1222 (m), 1175 (w), 1117 (w), 1087 (w), 1071 (w), 1034 (w) and 980 (w). Uv/Vis λ_{Max}(Toluene)/nm 320.

Nonacarbonyl (1,1-*bis*-(4-methoxyphenyl)methanimine) dimanganese(0) (5)

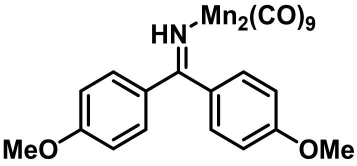

To a round-bottom flask equipped with a magnetic stirrer was added 1,1-*bis*-(4-methoxyphenyl)methanimine (1 eq., 1 mmol, 241 mg), [$Mn_2(CO)_{10}$] (1 eq., 1 mmol, 389 mg) and methylene chloride (40 mL). Trimethylamine N-oxide (1 eq., 1 mmol, 80 mg) was added in 5 mg portions. The solution was left stirring at ambient temperature for 1 hour, before being filtered through an alumina plug. The plug was washed with hexane (50 mL), then Et_2O (50 mL). The Et_2O fraction was reduced in *vacuo* and the resulting solid was recrystalised from boiling hexane, giving orange crystals (201 mg, 33%).

^1H NMR (500 MHz, CD_2Cl_2) δ 7.27 (2H, m), 7.05 (2H, m), 6.99 (2H, m), 6.81 (2H, m), 3.89 (3H, s), 3.81 (3H, s). ^{13}C NMR (CD_2Cl_2, 125 MHz) δ 186.01, 161.75, 160.61, 130.38, 130.33, 128.77, 128.47, 113.25, 112.98, 52.66, 52.23. LIFDI- MS m/z (ion, %): 602.96135 ($[M]^+$, 100). IR (toluene, cm^{-1}): 3087 (w), 3063 (w), 2084 (s), 2019 (s), 1998 (s), 1980 (s), 1958(s), 1929 (s), 1717 (m), 1603 (m), 1360 (w), 1218 (w), 1082 (w), 1030 (w). Uv/Vis λ_{Max}(Toluene)/nm 348 and 440sh.

Tetracarbonyl (η^2-2-(*p*-anisylC=NH)-5-methoxyphenyl) manganese(I) (6)

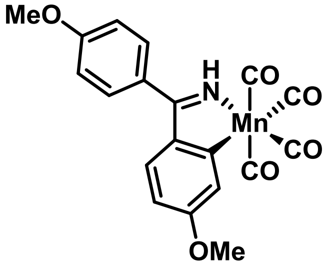

To an oven dried Schlenk tube equipped with a magnetic stirrer, under an atmosphere of N_2 was added $BnMn(CO)_5$ (1 eq., 1.8 mmol, 510 mg) and 1,1-*bis*-(4-methoxyphenyl) methanimine (1 eq., 1.8 mmol, 430 mg). The Schlenk tube was evacuated and backfilled with N_2 five times, then anhydrous, deoxygenated toluene was added. The solution was then heated to reflux for 6 hr and the Schlenk tube was covered in aluminium foil to exclude light. Reaction progress was monitored by solution phase IR, using the metal carbonyl stretching bands. When the reaction was complete, the reaction mixture was allowed to cool to room temperature and solvent removed in *vacuo*. The crude solid underwent purification using flash column chromatography on silica gel (4:1 v/v pet ether: methylene chloride to yield a pale yellow powder (560 mg, 76%).

^{1}H NMR (CD_2Cl_2, 500 MHz) δ (ppm) = 7.74 (1H, s), 7.61 (1H, s, *J* = 2.5 Hz), 7.48 (1H, d, *J* = 8.5 Hz), 7.41 (2H, m), 7.00 (2H, m), 6.62 (1H, dd, *J* = 1, 8.5 Hz), 3.92 (3H, s), 3.88 (3H, s). ^{13}C NMR (CD_2Cl_2, 125 MHz) δ (ppm) = 188.55, 161.50, 159.02, 132.12, 129.95, 129.90, 129.21, 125.79, 123.19, 123.19, 114.32, 109.93, 55.63, 55.37. R_f 0.2 (4:1 *v/v* pet ether: methylene chloride). LIFDI-MS m/z (ion, %): 407.01860 (100%, $[M]^+$). IR (Hexane, cm^{-1}): 3320 (w), 3098 (w), 3076 (w), 3040 (w), 3028 (w), 3012 (w), 2636 (w), 2882 (w), 2746 (w), 2340 (w), 2360 (w), 2072 (s), 1986 (s), 1978 (s), 1932 (s), 1606 (m), 1577 (m), 1536 (w), 1505 (w), 1495 (w), 1456 (w), 1420 (w), 1390 (w), 1296 (w), 1238 (w), 1176 (w), 1072 (w) and 1037 (w). Uv/Vis λ_{Max}(toluene)/ nm 339.

Benzyl pentacarbonyl manganese(I)

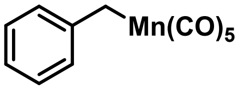

To an oven dried Schlenk tube equipped with a magnetic stirrer bar, under nitrogen, was added mercury (18 mL). Sodium metal (4 eq., 64.2 mmol, 1.78 g) was added in small pieces with high stirring to allow dissolution. In a separate Schlenk tube under nitrogen was added $Mn_2(CO)_{10}$ (1eq., 16.08 mmol, 6.24 g), followed by anhydrous, deoxygenated THF (100 mL). The THF solution was then transferred by cannula on to the sodium amalgam and was stirred for 3 hours. In a separate Schlenk tube equipped with a magnetic stirrer under nitrogen was added benzyl chloride (2 eq., 32.1 mmol, 3.7 mL). The Schlenk tube containing benzyl chloride was placed in a bath of ice and water and was put under vacuum with stirring for 60 seconds, before being backfilled with N_2. At ambient temperature, the THF solution of $NaMn(CO)_5$ was transferred by cannula into the benzyl chloride. The mixture was stirred at ambient temperature for 20 hours. The solution was then filtered through a bed of Celite®, and was washed with diethyl ether (5 × 40 mL). The contents were then loaded on to silica gel and this was added onto a pad of silica (5 cm). The pad was washed with pet ether (3 × 80 mL). The solvent was removed to yield the product containing benzyl chloride. Benzyl chloride was removed at 35 °C under vacuum. The product must be broken up with a spatula and put back under vacuum. A slightly yellow crystalline product was obtained (6.4 g, 69 %).

m.p. = 38.2-38.6 ^{o}C. ^{1}H NMR ($CDCl_3$, 500 MHz)δ (ppm) 2.42 (2H, s), 6.98 (1H, m), 7.19 (4H, m); ^{13}C NMR ($CDCl_3$, 125 MHz) δ 11.25, 123.61, 125.98, 127.74, 128.77, and 151.95, 212.46. R_f 0.37 (pet ether). LIFDI-MS m/z (ion, %): 285.96677([M]$^+$, 100).IR (Hexane, cm^{-1}): 2106 (s), 2042 (s), 2010 (s), 1989 (S), 1598 (w), 1465 (w), 1379 (w), 1261 (w) and 1220 (w).

***fac*-Tricarbonyl trisacetonitrile manganese(I) hexafluorophosphate**

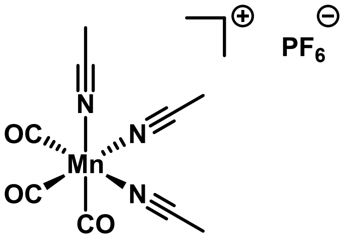

An oven dried Schlenk flask equipped with a magnetic stirrer was evacuated and backfilled with N_2 five times. [$Mn_2(CO)_{10}$] (1 eq., 10 mmol, 3.9 g) was dissolved in anhydrous deoxygenated MeCN (90 mL) and $NOPF_6$ (2 eq., 20 mmol, 5 g) was added. The reaction mixture was stirred at ambient temperature for 1 hour, reduced to approx. 30 mL, heated at 50 °C for 24 hr and solvent removed to afford a pale-yellow solid (1.1 g, 20%). Crude material was used immediately in the subsequent React IR™ experiment.

IR (MeCN, cm^{-1}): 2065 (s), 1975 (s).

[$Mn_7(\mu_3\text{-}OH)_8(CO)_{18}$]

To a 50 ml round-bottom flask under ambient conditions was added $Mn_2(CO)_{10}$ (195 mg, 0.5 mmol, 1 eq.), trimethylamine *N*-oxide (333 mg, 3 mmol, 6 eq.), benzophenone (78 mg, 0.4 mmol, 0.8 eq.) and THF (10 ml). The solution was stirred for 18 hours at room temperature, before the solvent was removed in vacuo. The crude material was recrystallized from $CHCl_3$/hexane to yield a yellow powder (98 mg, 67%).

IR (KBr-disc, cm^{-1}): 3632 (w), 3269 (w), 2041 (s), 2021 (s), 1908 (s), 1479 (w), 952 (w), 765 (w), 691 (w), 645 (w), 513 (w)

IR (Toluene, cm^{-1}): 2017 (s) cm^{-1}, 1905 (s).

3. NMR Spectra

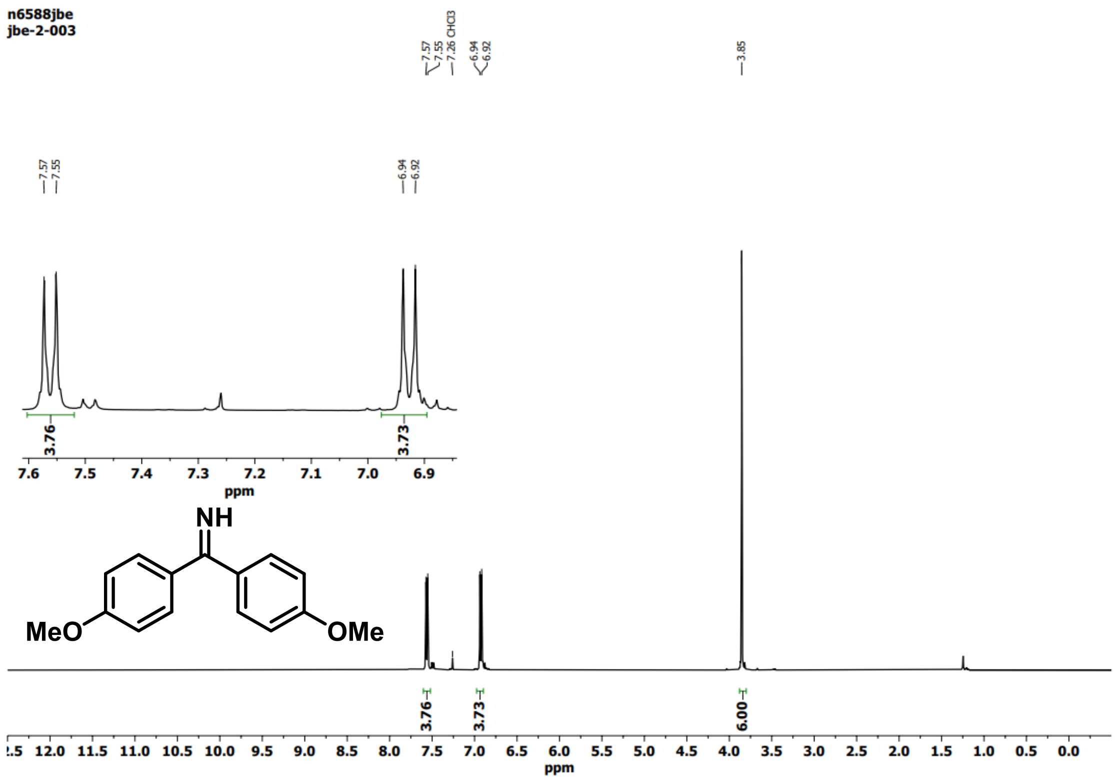

Figure S1. ^{1}H NMR spectrum of compound **1** in deuterated chloroform.

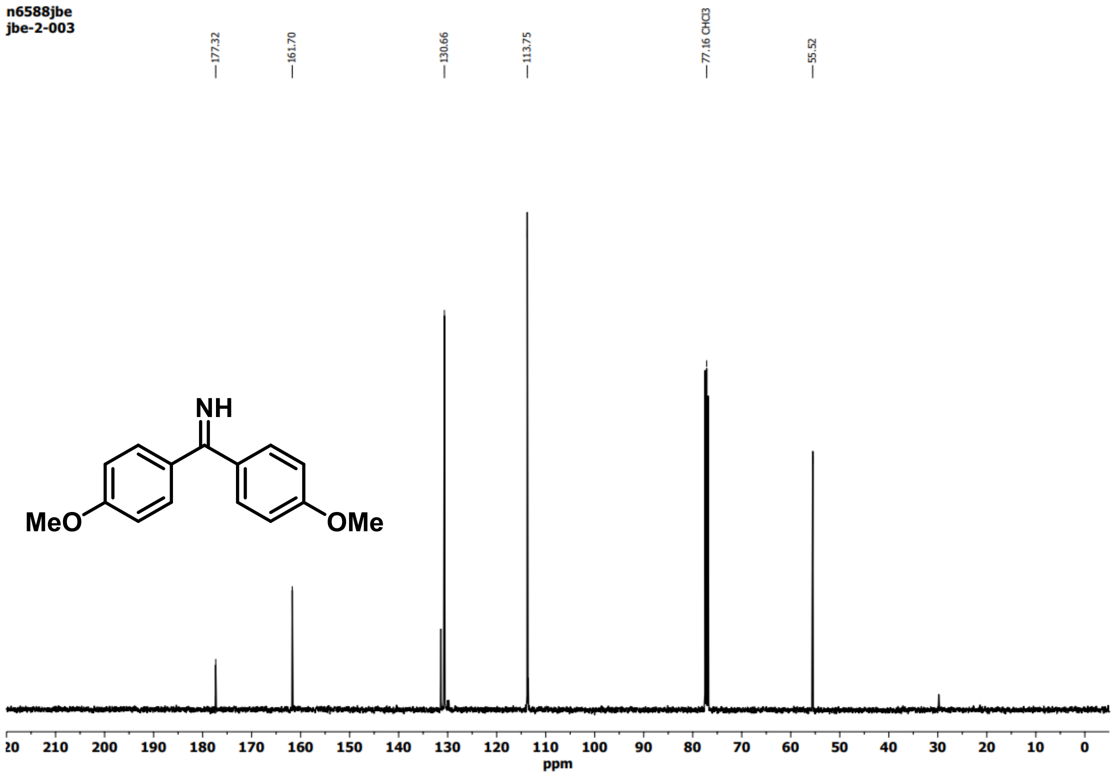

Figure S2. ^{13}C NMR spectrum of compound **1** in deuterated chloroform.

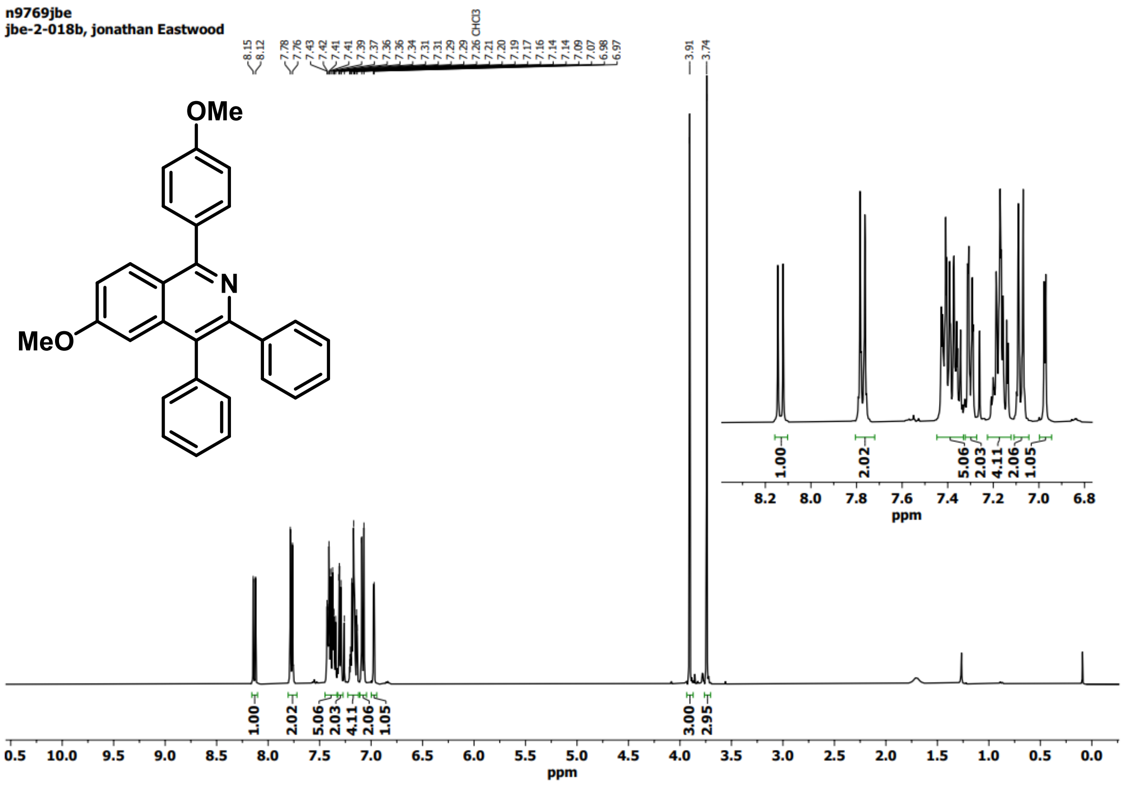

Figure S3. ^{1}H NMR spectrum of compound **3** in deuterated chloroform.

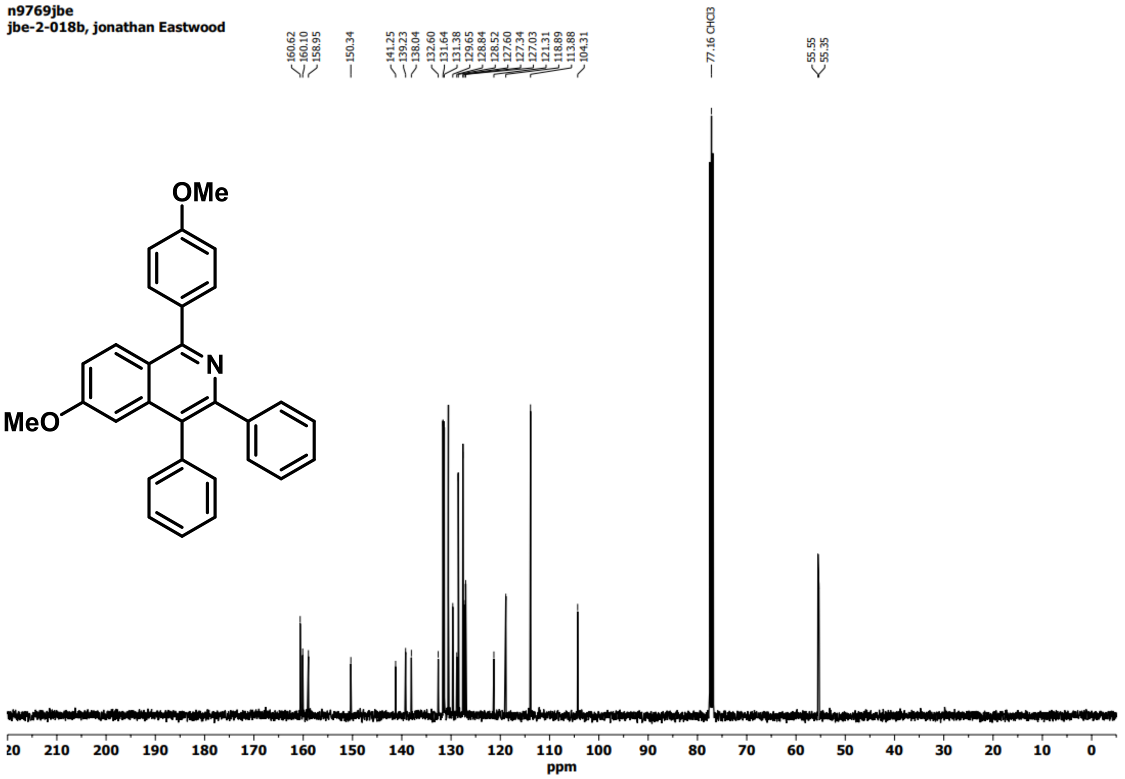

Figure S4. ^{13}C NMR spectrum of compound **3** in deuterated chloroform.

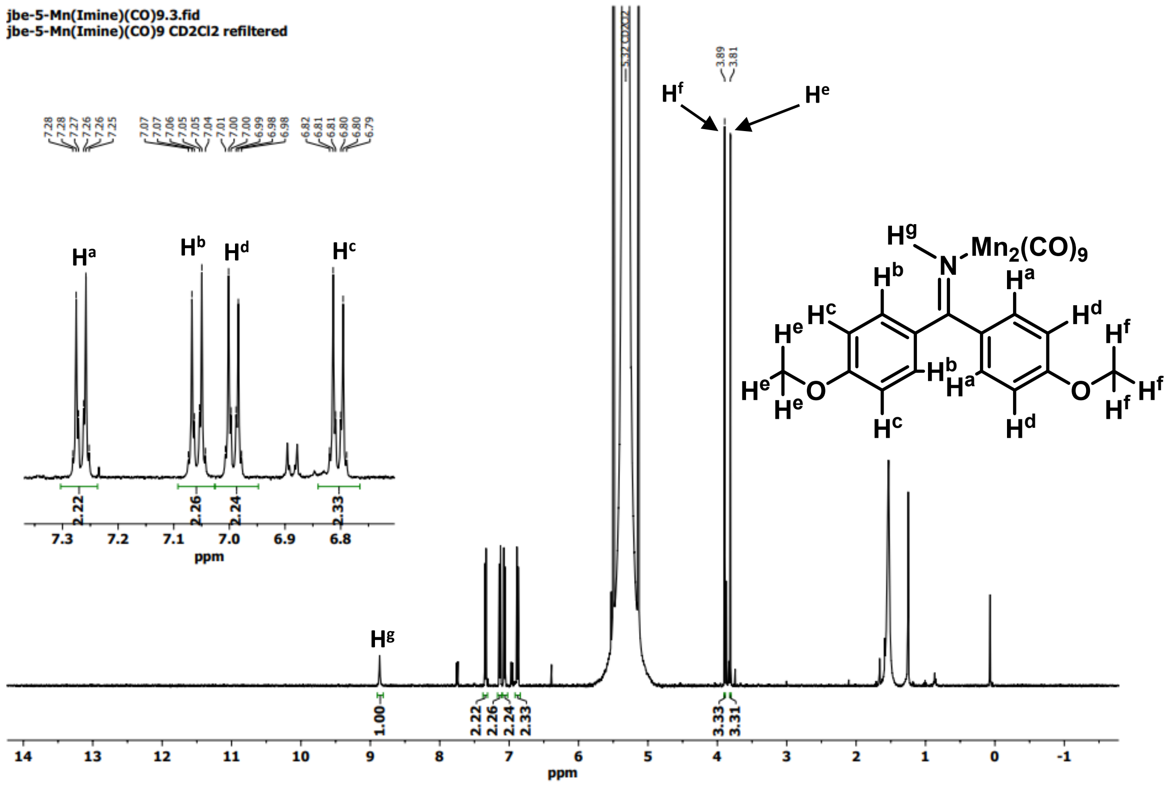

Figure S5. ^{1}H NMR spectrum of compound **5** in deuterated methylene chloride.

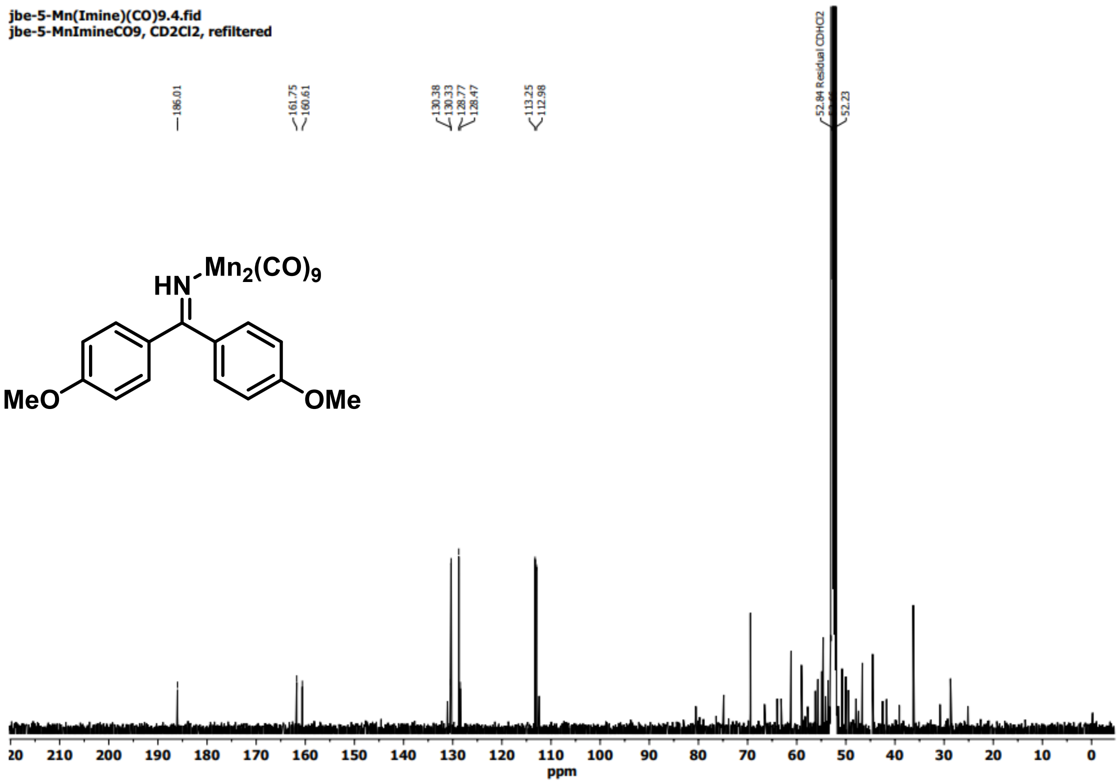

Figure S6. ^{13}C NMR spectrum of compound **5** in deuterated methylene chloride.

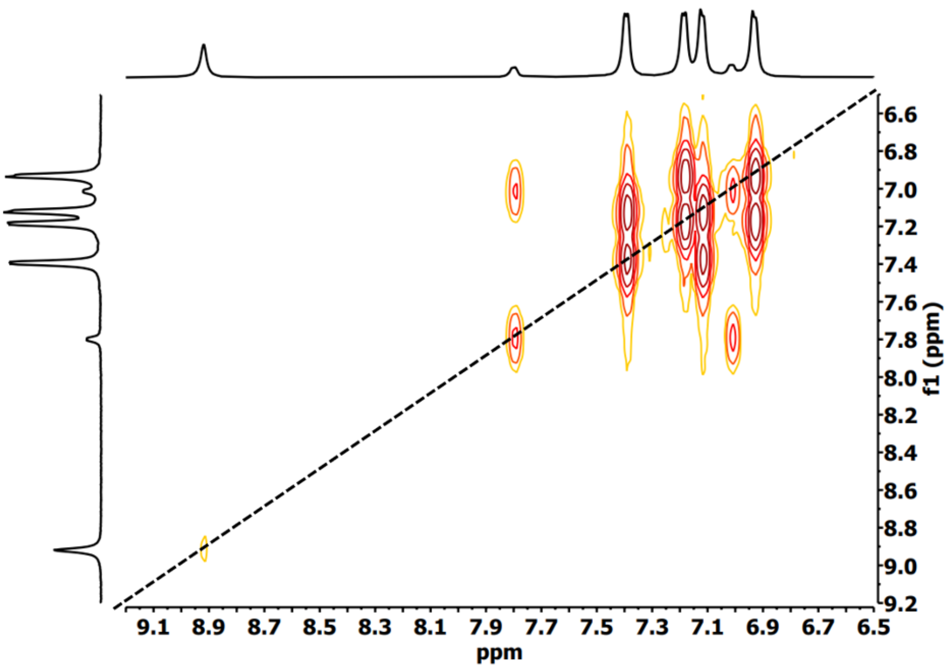

Figure S7. ^{1}H ^{1}H COSY NMR spectrum of compound **5** in deuterated methylene chloride between 9.2 and 6.5 ppm.

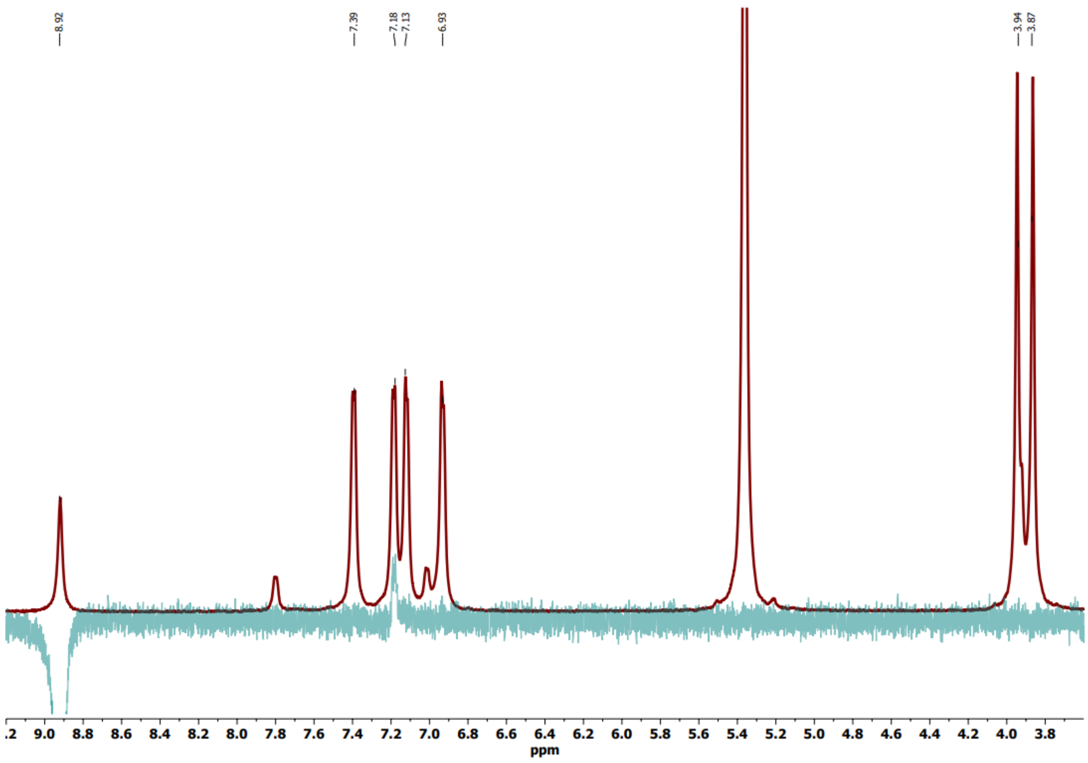

Figure S8. ^{1}H NMR spectrum of compound **5** in deuterated methylene chloride (Red) and 1D NOESY spectrum centred at 8.92 ppm between 9.2 and 3.6 ppm.

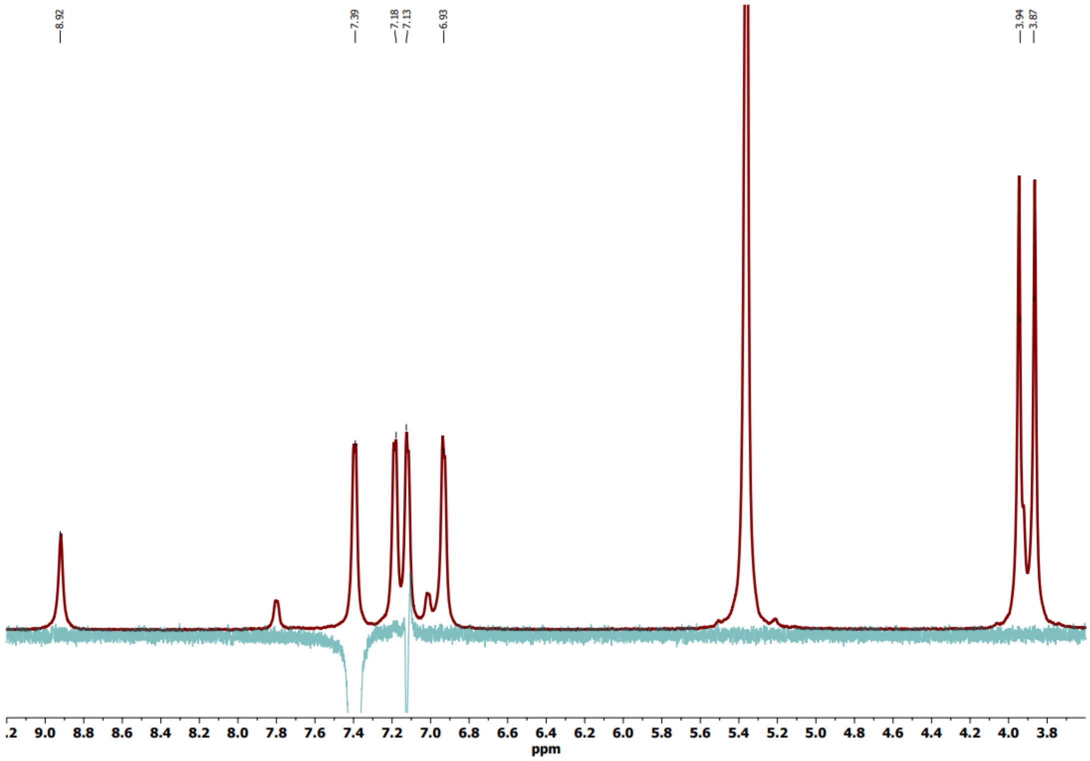

Figure S9. ^{1}H NMR spectrum of compound **5** in deuterated methylene chloride (Red) and 1D NOESY spectrum centred at 7.39 ppm between 9.2 and 3.6 ppm.

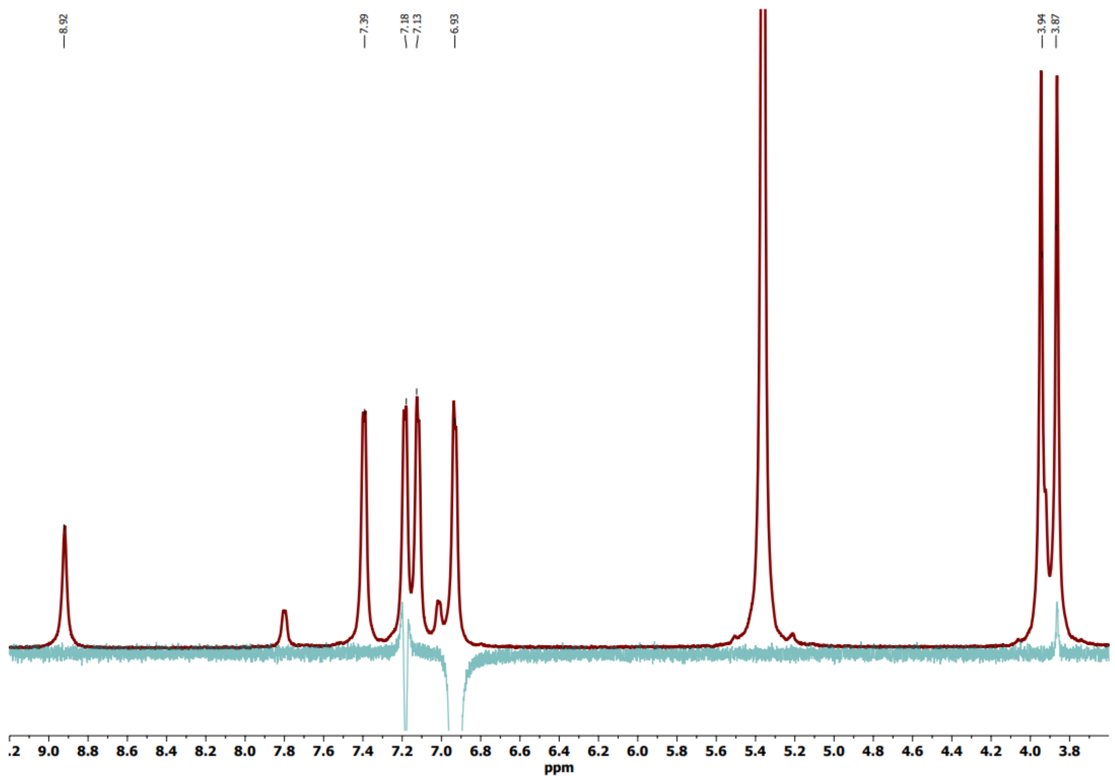

Figure S10. ^{1}H NMR spectrum of compound **5** in deuterated methylene chloride (Red) and 1D NOESY spectrum centred at 6.93 ppm between 9.2 and 3.6 ppm.

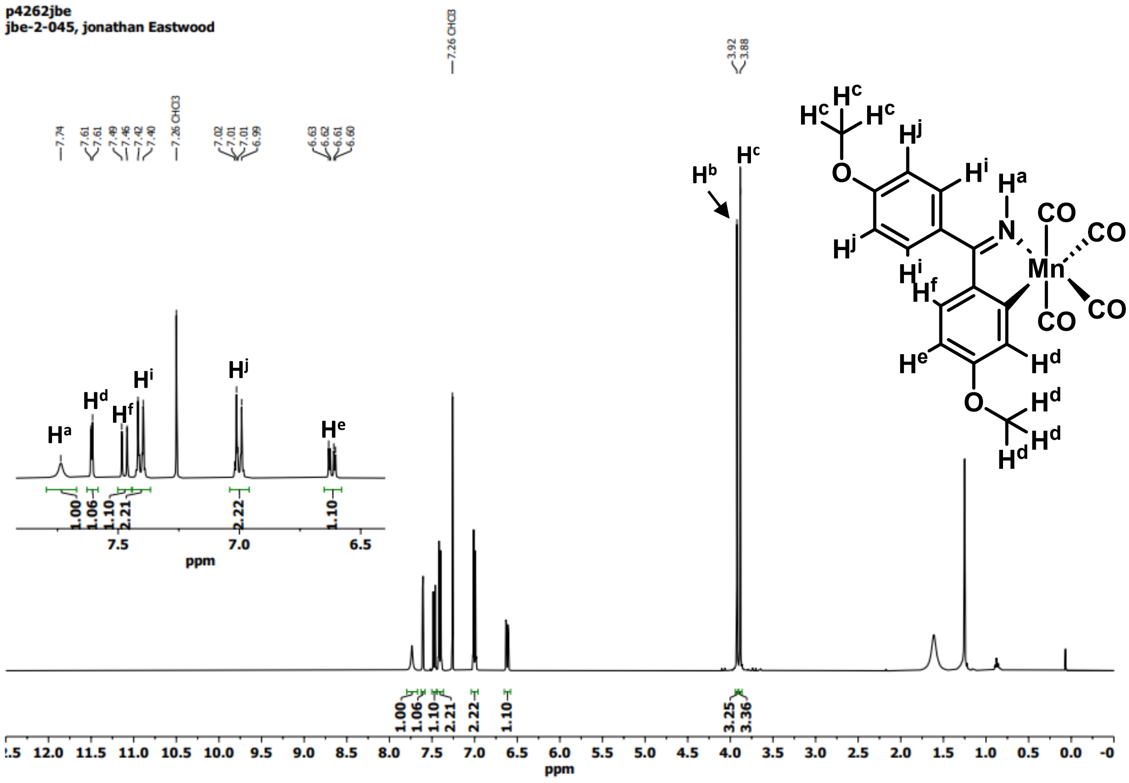

Figure S11. ^{1}H NMR spectrum of compound **6** in deuterated chloroform.

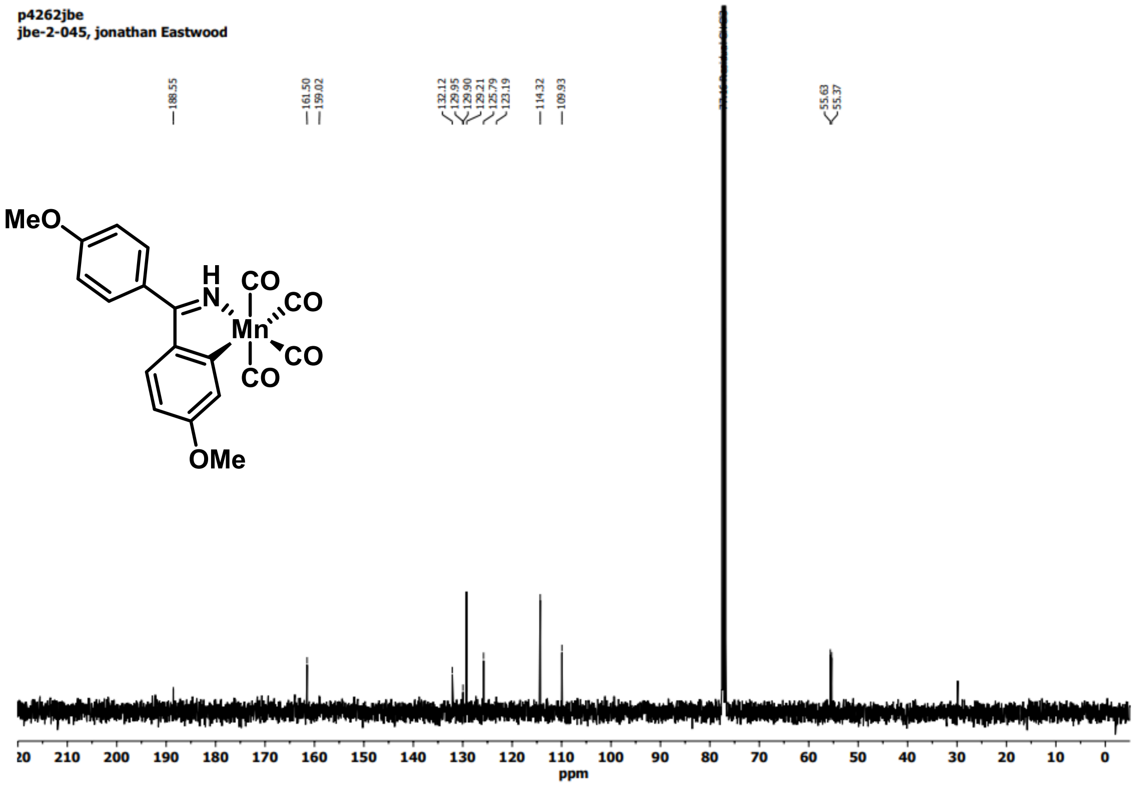

Figure S12. ^{13}C NMR spectrum of compound **6** in deuterated chloroform.

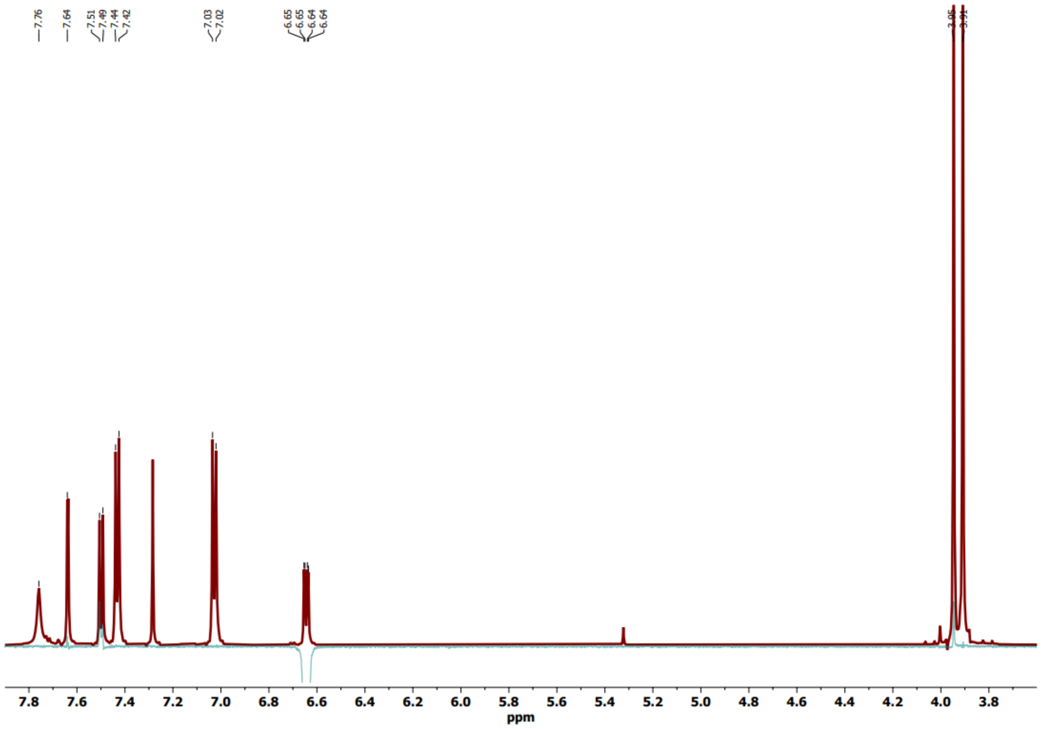

Figure S13. ^{1}H NMR spectrum of compound **6** in deuterated chloroform chloride (Red) and 1D NOESY spectrum centred at 6.64 ppm between 7.9 and 3.6 ppm.

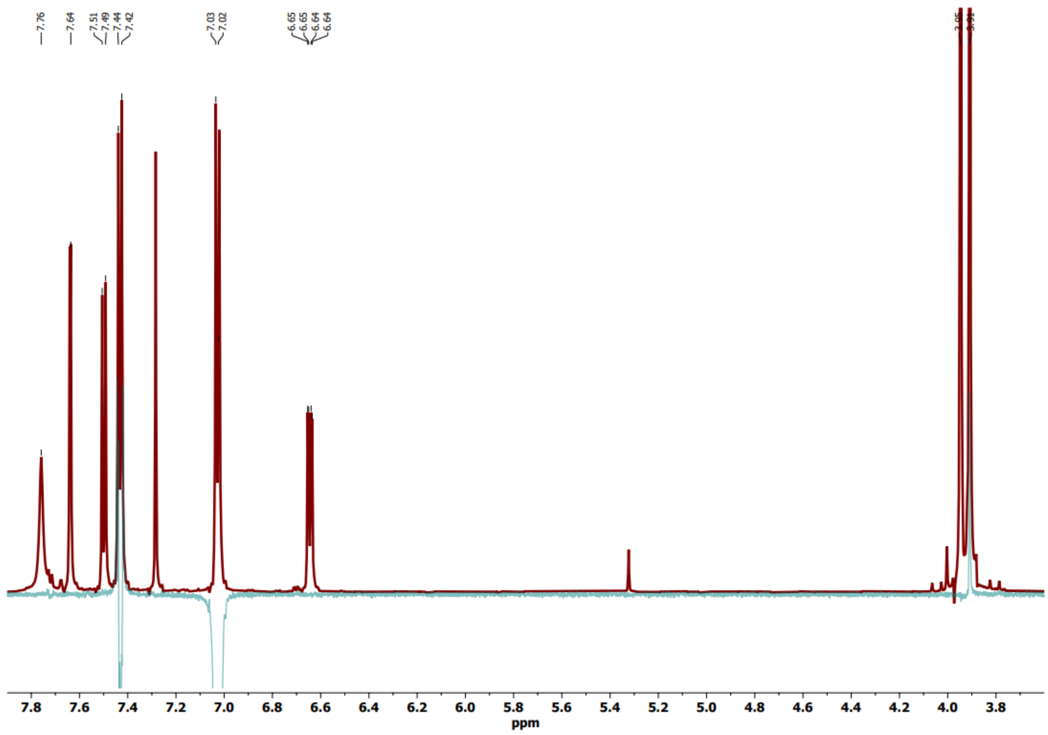

Figure S14. ^{1}H NMR spectrum of compound **6** in deuterated chloroform chloride (Red) and 1D NOESY spectrum centred at 6.64 ppm between 7.9 and 3.6 ppm.

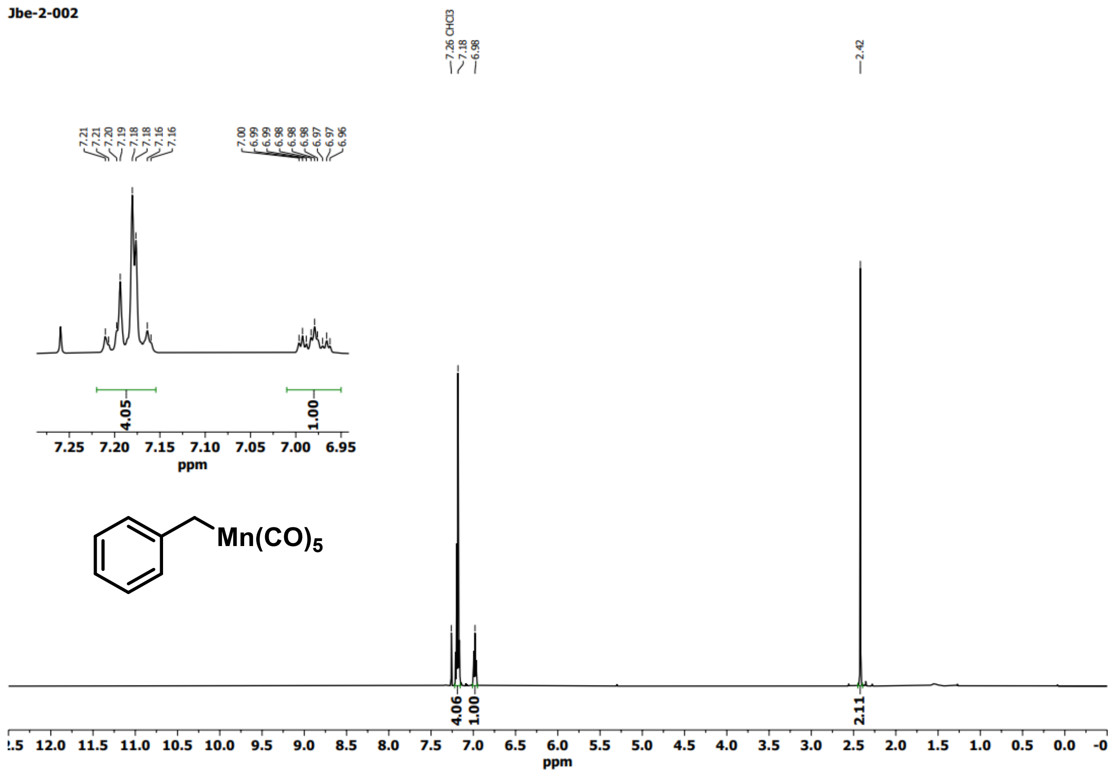

Figure S15. ^{1}H NMR spectrum of compound **8** in deuterated chloroform.

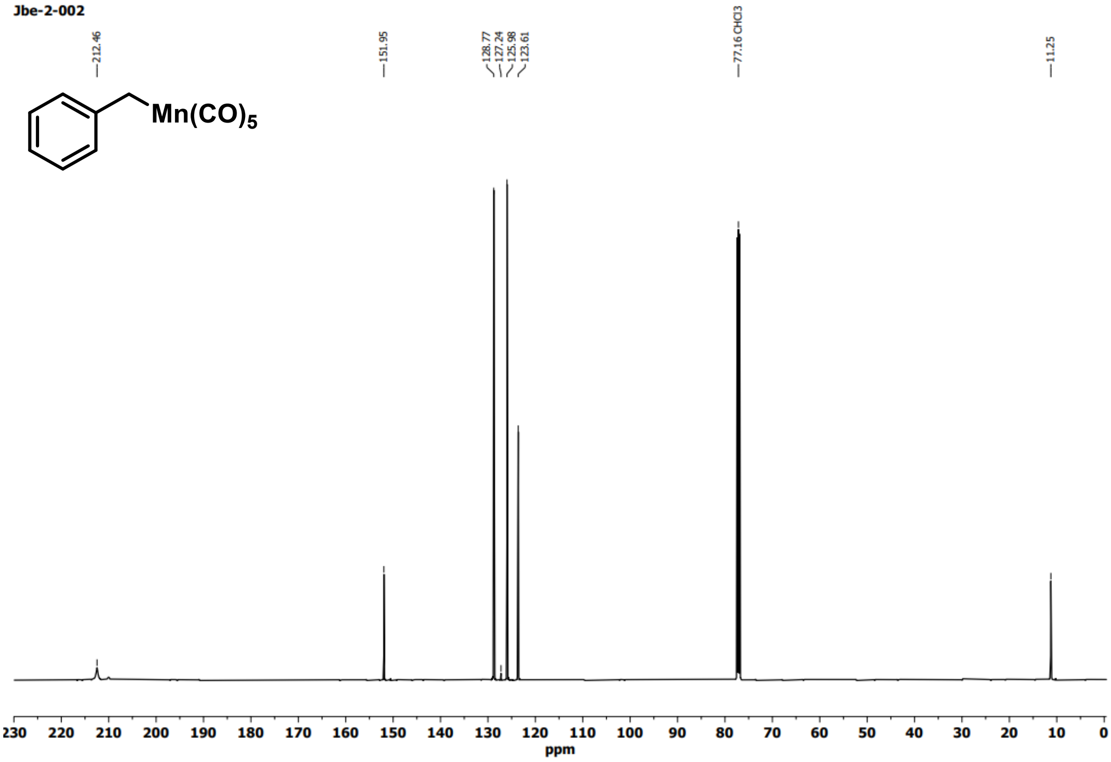

Figure S16. ^{13}C NMR spectrum of compound **8** in deuterated chloroform.

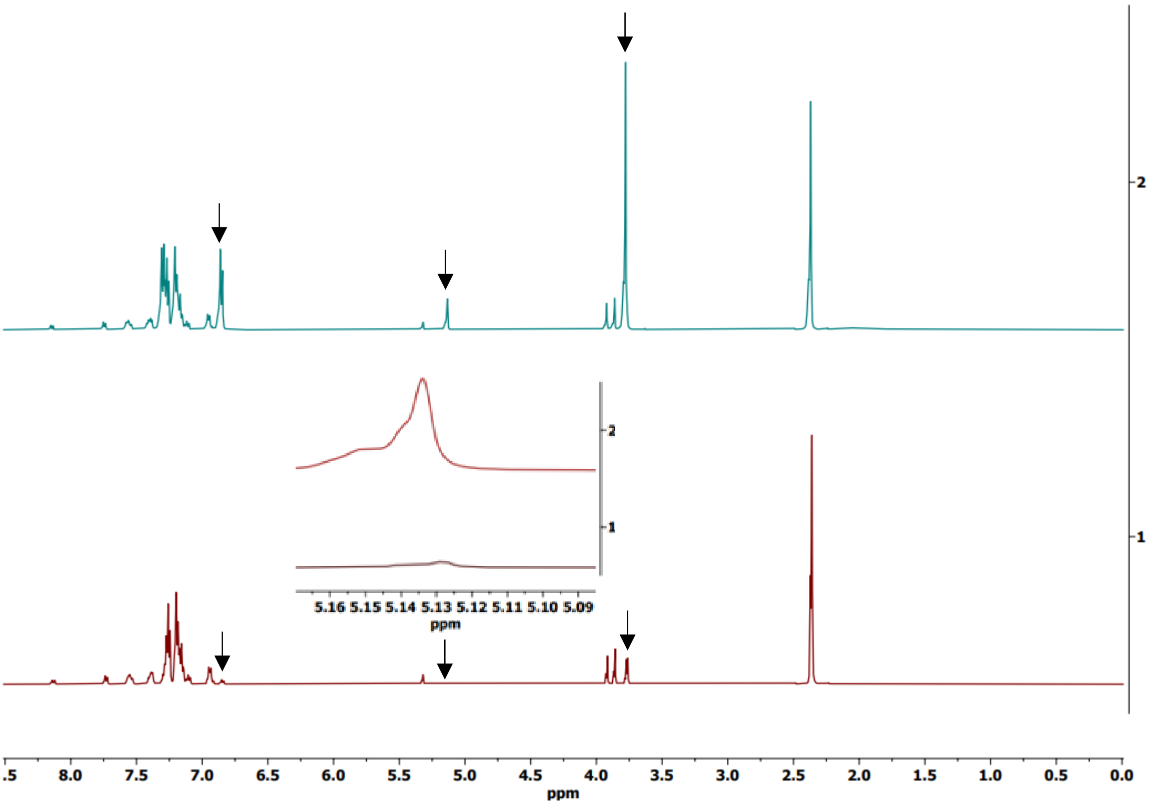

Figure S17. **1**) ^{1}H NMR spectrum of a reaction aliquot taken from the reaction between imine **1** and Ph_2C_2 catalysed by $[Mn_2(CO)_{10}]$. **2**) Aliquot from **1**) spiked with 1,1-*bis*-(4-methoxyphenyl)methanamine. 1,1-*bis*-(4-methoxyphenyl)methanamine peaks denoted by black arrows.

4. IR Spectra

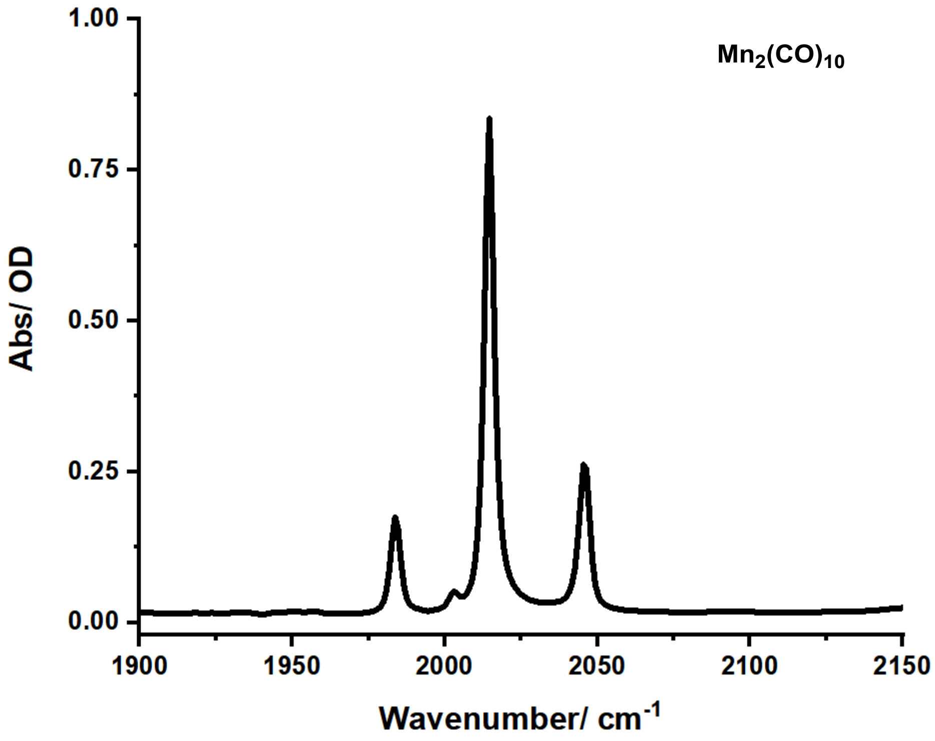

Figure S18. IR spectrum of $\mathbf{Mn_2(CO)_{10}}$ in toluene.

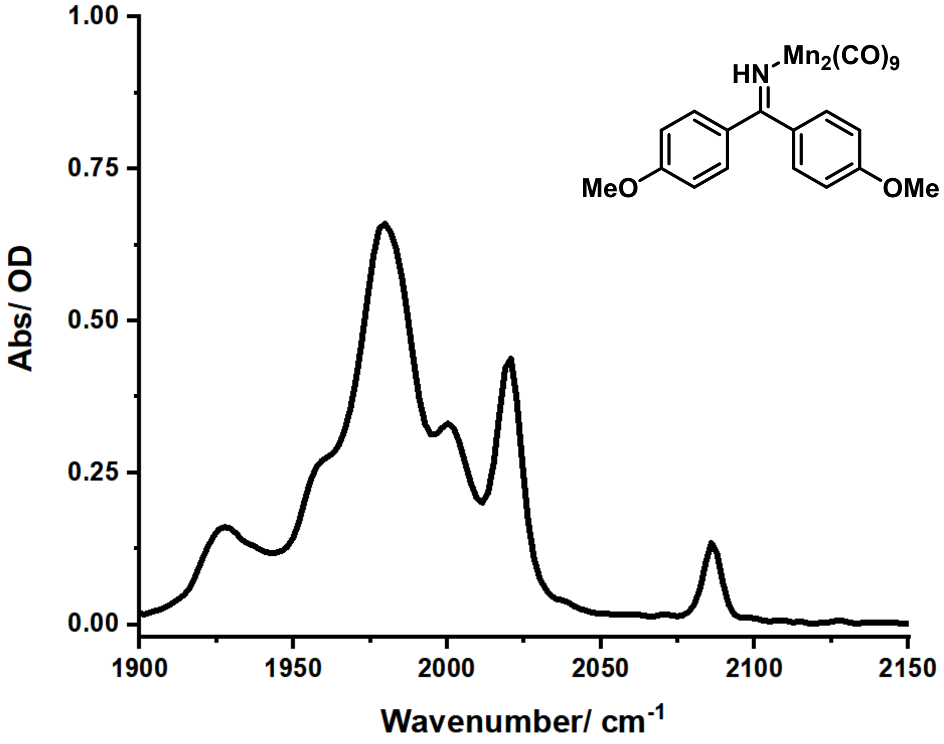

Figure S19. IR spectrum of compound **5** in toluene.

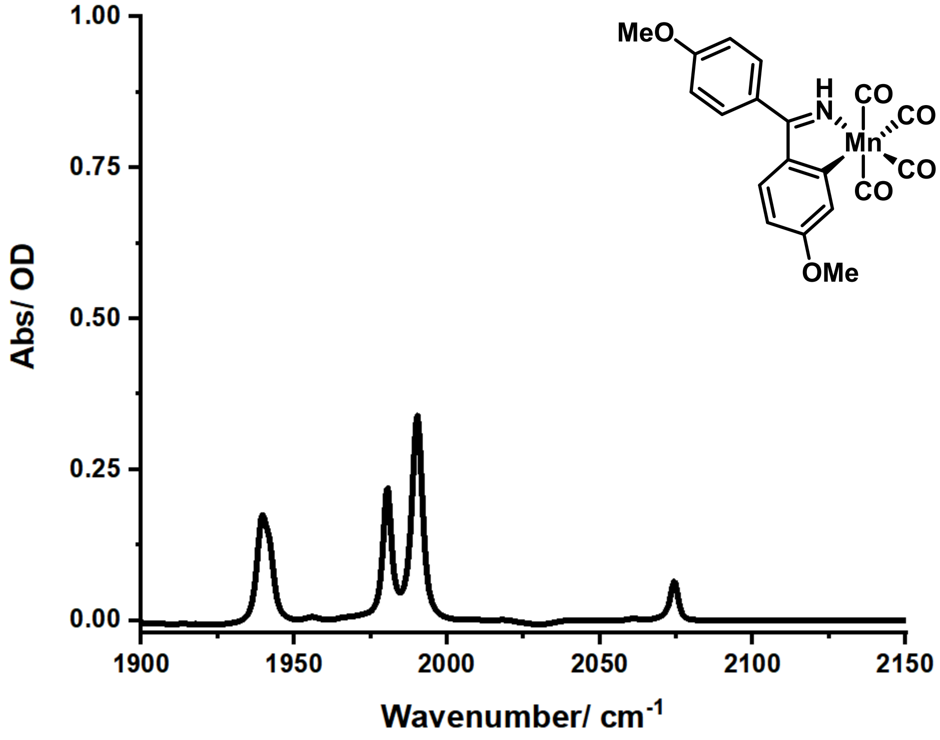

Figure S20. IR spectrum of compound **6** in toluene.

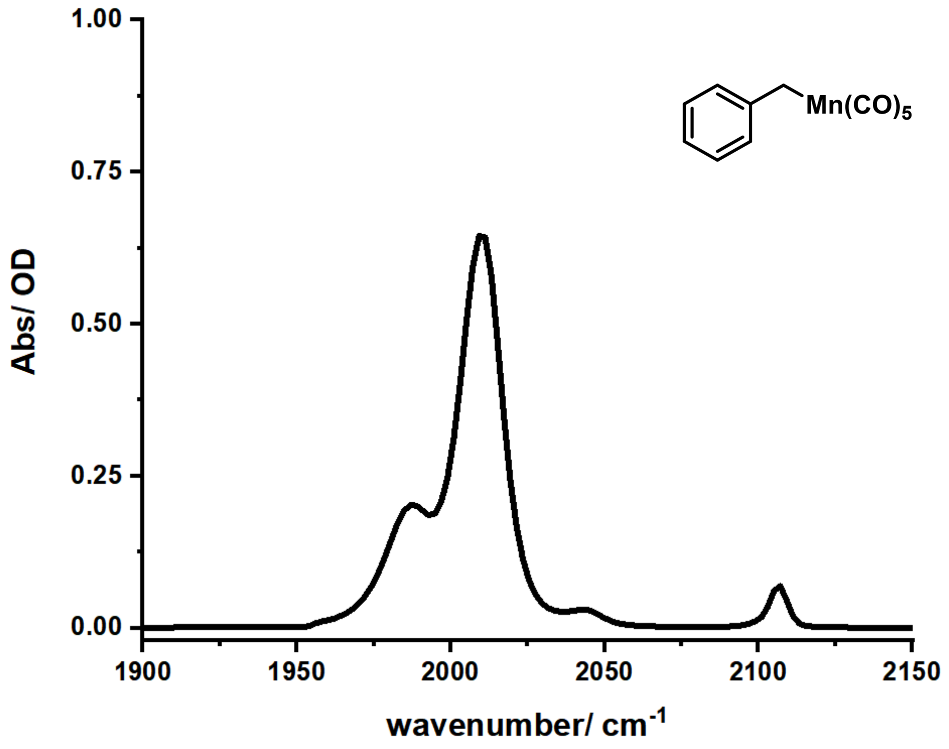

Figure S21. IR spectrum of compound **8** in toluene.

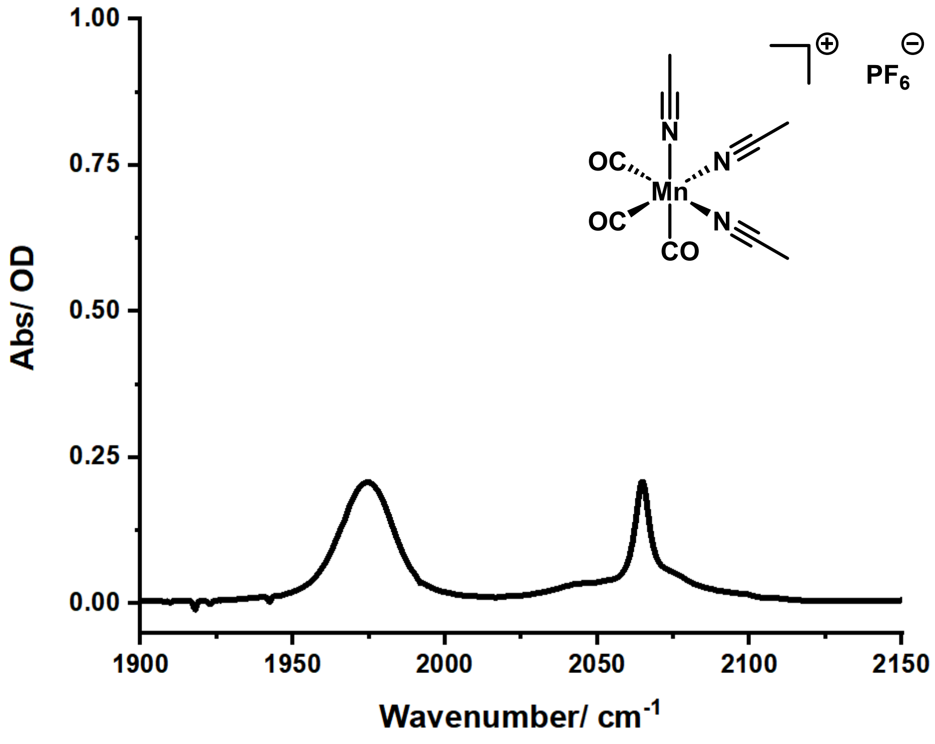

Figure S22. IR spectrum of compound **9** in MeCN.

5. Uv-Vis Spectra of $Mn_2(CO)_{10}$ used in Pump-Probe TRIR experiments

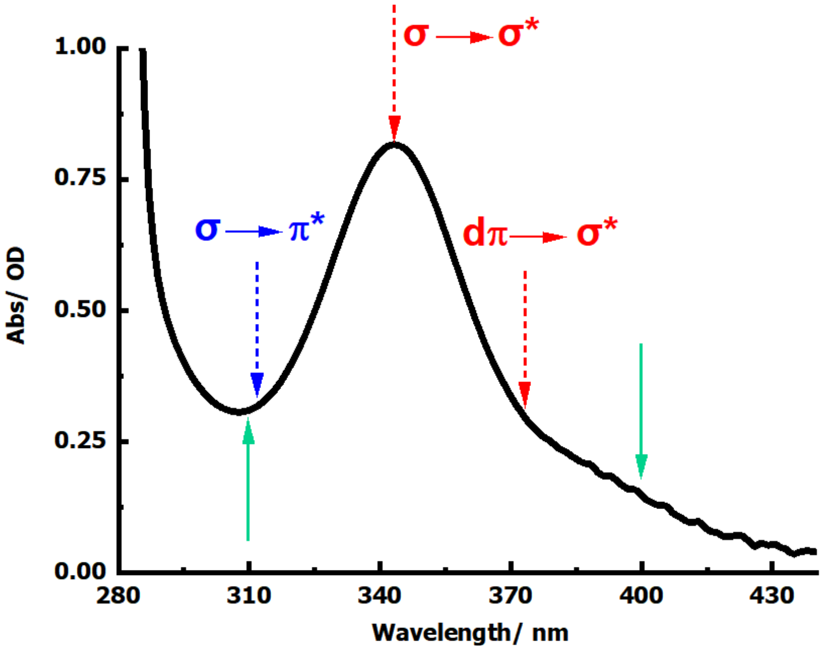

Figure S23. Uv-Vis spectrum of $\mathbf{Mn_2(CO)_{10}}$ in toluene. Blue arrows denote CO dissociative electronic transitions[7], Red arrows electronic transitions which result in homolytic Mn−Mn bond cleavage[7], and Green arrows are pump wavelengths used in TRIR experiments (310 and 400 nm).

6. Additional control TRIR experiments

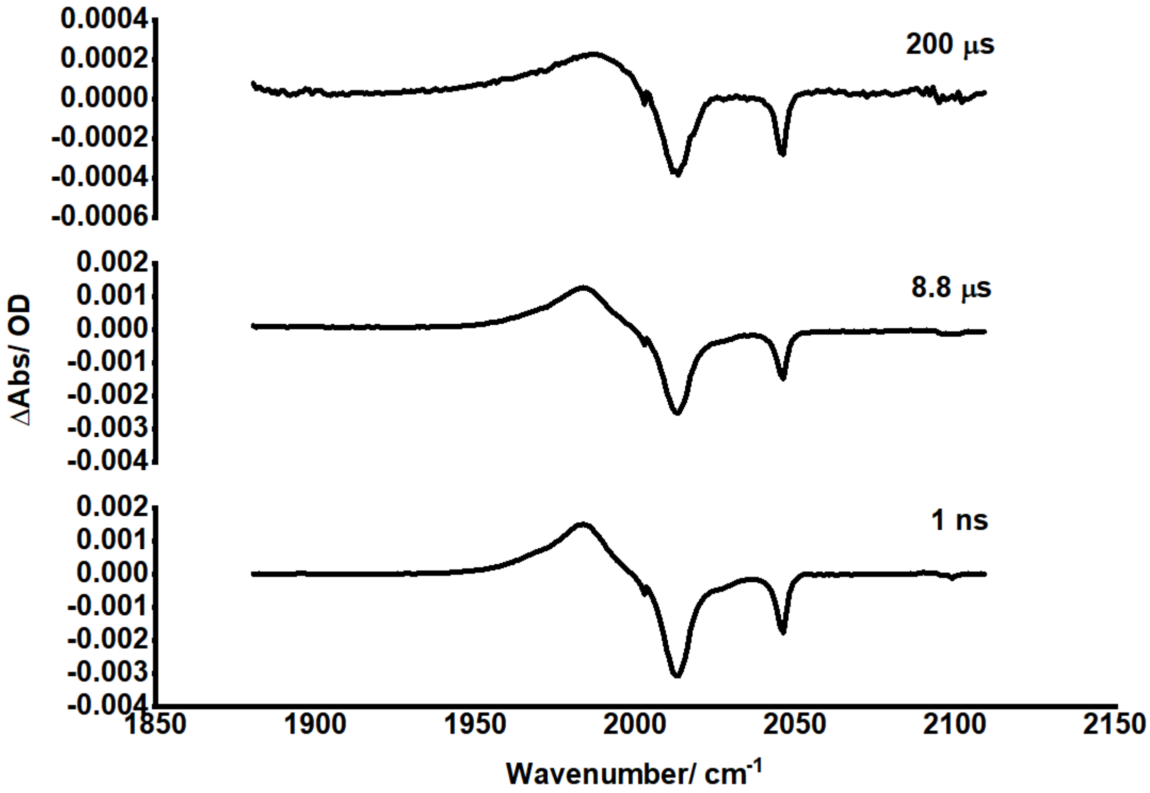

Figure S24. 7.7 mg $Mn_2(CO)_{10}$ in 15 mL PhC_2H with a 400 nm pump wavelength under an atmosphere of argon. Band at 1984 cm^{-1} formed for $Mn(CO)_5$. The transient intensity decreases over the course of *ca*. 200 µs as $Mn_2(CO)_{10}$ bleach bands recover, implying that PhC_2H, a less sterically hindered alkyne than Ph_2C_2 does not react with $Mn(CO)_5$.

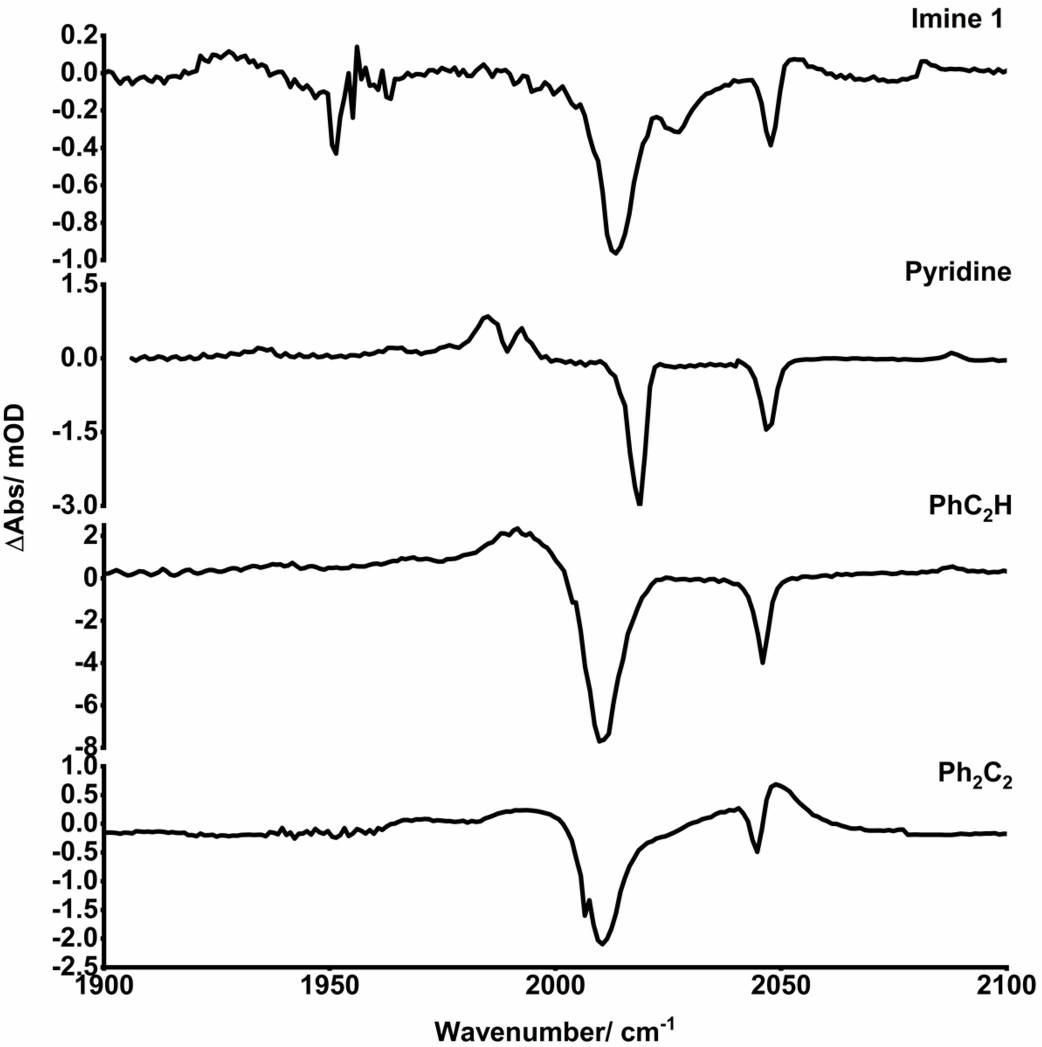

Figure S25. $Mn_2(CO)_{10}$ in toluene solution excited at 310 nm with a pump-probe delay of 20 µs in the presence of different ligands. In the case of Imine **1**, pyridine and the less sterically hindered alkyne, phenyl acetylene, a high energy band at approximately 2085 cm^{-1} was observed. This is indicative of $Mn_2(CO)_9L$ complexes. In the instance of diphenyl acetylene, no evidence of a high energy band for the formation of a $Mn_2(CO)_9L$ complex was ever observed.

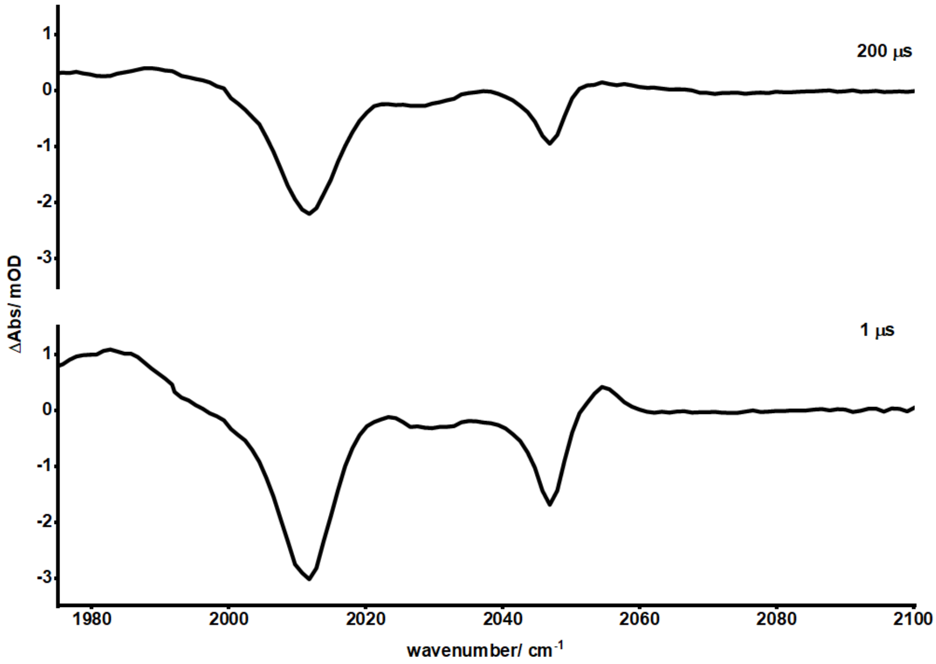

Figure S26. $Mn_2(CO)_{10}$ in toluene solution excited at 310 nm with a pump-probe delay of 200 μs (top) and 1 μs(bottom) to determine the % of bleach recovery 2011 cm^{-1} and ergo, the proportion of Mn–Mn and Mn–CO bond cleavage.

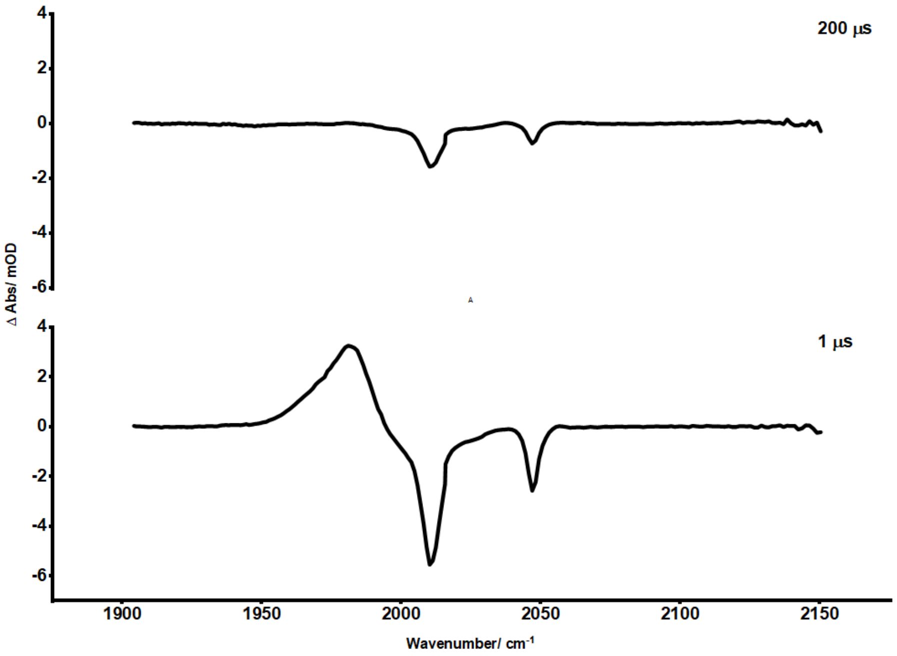

Figure S27. $Mn_2(CO)_{10}$ in toluene solution excited at 400 nm with a pump-probe delay of 200 μs (top) and 1 μs (bottom) to determine the % of bleach band recovery at 2011 cm^{-1} and ergo, the proportion of Mn–Mn and Mn–CO bond cleavage.

7. X-ray data

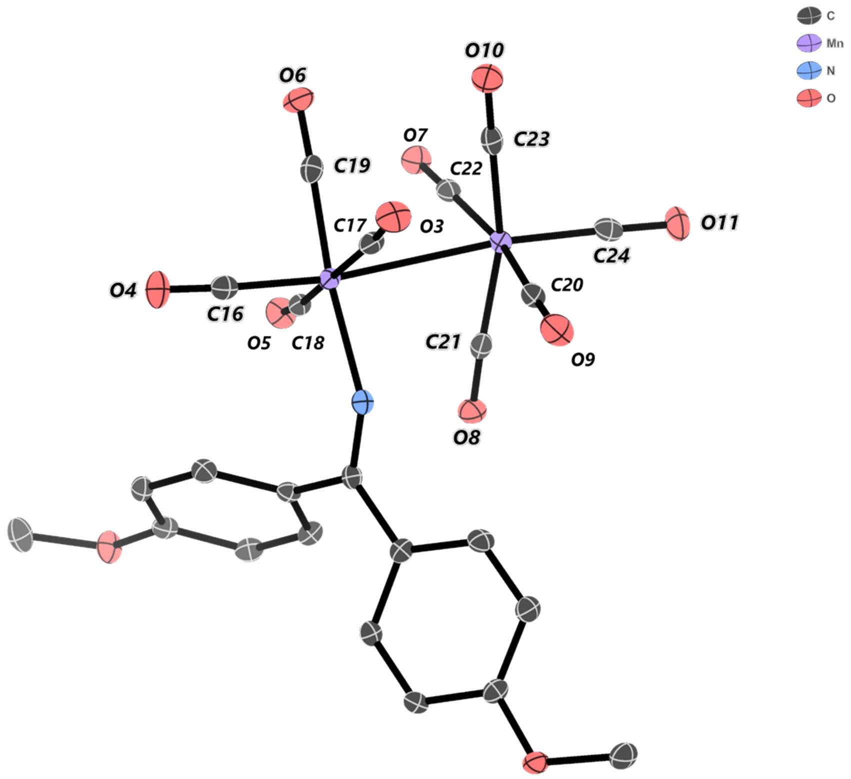

Figure S28. Single crystal X-ray diffraction structure of **6**. Thermal ellipsoids shown with 50% probability and hydrogen atoms removed for clarity.

Table S1 Crystal data and structure refinement for 6.

CCDC code	2334024
Empirical formula	$C_{24}H_{15}Mn_2NO_{11}$
Formula weight	603.25
Temperature/K	110.00(14)
Crystal system	triclinic
Space group	P-1
a/Å	9.0789(5)
b/Å	11.2974(8)
c/Å	12.7130(8)
α/°	110.200(6)
β/°	94.410(5)
γ/°	92.531(5)
Volume/Å^3	1216.63(14)
Z	2
ρ_{calc}g/cm^3	1.647
μ/mm^{-1}	1.102
F(000)	608.0
Crystal size/mm^3	0.41 × 0.292 × 0.224
Radiation	Mo Kα (λ = 0.71073)
2Θ range for data collection/°	7.114 to 58.046
Index ranges	-12 ≤ h ≤ 11, -15 ≤ k ≤ 14, -17 ≤ l ≤ 16
Reflections collected	9897
Independent reflections	5548 [R_{int} = 0.0248, R_{sigma} = 0.0481]
Data/restraints/parameters	5548/0/349
Goodness-of-fit on F^2	1.063
Final R indexes [I>=2σ (I)]	R_1 = 0.0355, wR_2 = 0.0734
Final R indexes [all data]	R_1 = 0.0456, wR_2 = 0.0804
Largest diff. peak/hole / e Å^{-3}	0.44/-0.41

8. References

[1] W. C. Still, M. Kahn and A. Mitra, *J. Org. Chem.* **1978**, *43*, 2923–2925.

[2] O. v Dolomanov, L. J. Bourhis, R. J. Gildea, J. A. K. Howard and H. Puschmann, *J. Appl. Cryst.* **2009**, *42*, 339–341.

[3] G. M. Sheldrick, *Acta Crystallogr.* **2015**, *A71*, 3–8.

[4] G. M. Sheldrick, *Acta Crystallogr.* **2015**, *C71*, 3–8.

[5] G. M. Greetham, P. M. Donaldson, C. Nation, I. V. Sazanovich, I. P. Clark, D. J. Shaw, A. W. Parker and M. Towrie, *Appl. Spectrosc.* **2016**, *70*, 645–653.

[6] G. M. Greetham, D. Sole, I. P. Clark, A. W. Parker, M. R. Pollard and M. Towrie, *Rev. Sci. Instrum.* **2012**, *83*, 103107.

[7] H. Cho, K. Hong, M. L. Strader, J. H. Lee, R. W. Schoenlein, N. Huse and T. K. Kim, *Inorg. Chem.* **2016**, *55*, 5895–5903.
